# Ad26.COV2.S priming provided a solid immunological base for mRNA-based COVID-19 booster vaccination

**DOI:** 10.1016/j.isci.2022.105753

**Published:** 2022-12-07

**Authors:** Daryl Geers, Roos S.G. Sablerolles, Debbie van Baarle, Neeltje A. Kootstra, Wim J.R. Rietdijk, Katharina S. Schmitz, Lennert Gommers, Susanne Bogers, Nella J. Nieuwkoop, Laura L.A. van Dijk, Eva van Haren, Melvin Lafeber, Virgil A.S.H. Dalm, Abraham Goorhuis, Douwe F. Postma, Leo G. Visser, Anke L.W. Huckriede, Alessandro Sette, Alba Grifoni, Rik L. de Swart, Marion P.G. Koopmans, P. Hugo M. van der Kuy, Corine H. GeurtsvanKessel, Rory D. de Vries, SWITCH research group

**Affiliations:** 1 Department of Viroscience, Erasmus Medical Center, Rotterdam, the Netherlands; 2 Department of Hospital Pharmacy, Erasmus Medical Center, Rotterdam, the Netherlands; 3 Department of Medical Microbiology and Infection Prevention, University Medical Center Groningen, University of Groningen, Groningen, the Netherlands; 4 Center for Infectious Disease Control, National Institute for Public Health and the Environment, Bilthoven, the Netherlands; 5 Department of Experimental Immunology, Amsterdam University Medical Centers, Amsterdam Infection and Immunity Institute, University of Amsterdam, Amsterdam, the Netherlands; 6 Department of Internal Medicine, Erasmus Medical Center, Rotterdam, the Netherlands; 7 Department of Internal Medicine, Division of Allergy & Clinical Immunology and Department of Immunology, Erasmus Medical Center, Rotterdam, the Netherlands; 8 Center of Tropical Medicine and Travel Medicine, Department of Infectious Diseases, Amsterdam University Medical Centers, Amsterdam, the Netherlands; 9 Infection & Immunity, Amsterdam Public Health, University of Amsterdam, Amsterdam, the Netherlands; 10 Department of Internal Medicine and Infectious Diseases, University Medical Center Groningen, Groningen, the Netherlands; 11 Department of Infectious Diseases, Leiden University Medical Center, Leiden, the Netherlands; 12 Center for Infectious Disease and Vaccine Research, La Jolla Institute for Immunology (LJI), La Jolla, CA, USA; 13 Department of Medicine, Division of Infectious Diseases and Global Public Health, University of California, San Diego (UCSD), La Jolla, La Jolla, CA, USA

**Keywords:** Health sciences, immunology, immune response, virology

## Abstract

The emergence of novel SARS-CoV-2 variants led to the recommendation of booster vaccinations after Ad26.COV2.S priming. It was previously shown that heterologous booster vaccination induces high antibody levels, but how heterologous boosters affect other functional aspects of the immune response remained unknown. Here, we performed immunological profiling of Ad26.COV2.S-primed individuals before and after homologous or heterologous (mRNA-1273 or BNT162b2) booster. Booster vaccinations increased functional antibodies targeting ancestral SARS-CoV-2 and emerging variants. Especially heterologous booster vaccinations induced high levels of functional antibodies. In contrast, T-cell responses were similar in magnitude following homologous or heterologous booster vaccination and retained cross-reactivity towards variants. Booster vaccination led to a minimal expansion of SARS-CoV-2-specific T-cell clones and no increase in the breadth of the T-cell repertoire. In conclusion, we show that Ad26.COV2.S priming vaccination provided a solid immunological base for heterologous boosting, increasing humoral and cellular responses targeting emerging variants of concern.

## Highlights

• mRNA-based booster after Ad26.COV2.S priming induces strong immune responses
• Booster vaccinations induce cross-reactive (non-)neutralizing antibodies
• T cells after booster vaccination retain reactivity with novel variants of concern
• Booster vaccinations do not increase the T-cell repertoire diversity

## Graphical abstract

**Figure:**
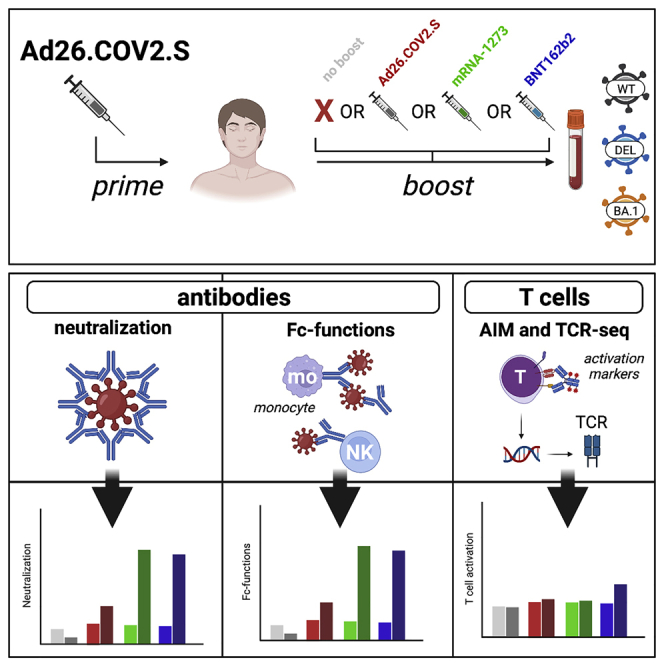

## Introduction

The emergence of severe acute respiratory syndrome coronavirus-2 (SARS-CoV-2) variants that are antigenically distinct and can evade vaccine-induced antibody responses^1^^,^^2^ resulted in the recommendation of COVID-19 booster vaccinations.^3^^,^^4^ Currently circulating variants are predominantly viruses from the Omicron sub-lineage. These variants harbor several mutations in the spike (S) protein that allow for partial immune escape at the antibody level. Previous studies have shown that mRNA-based booster vaccinations increase both S-specific antibodies and to a lesser extent T-cell responses, and restore clinical protection against severe disease after infection with antigenically distinct variants.^5^^,^^6^^,^^7^^,^^8^

According to the final evaluation of the phase 3 clinical trial, vaccination with a single dose of Ad26.COV2.S induces protection against moderate to severe-critical COVID-19, to varying degrees between different SARS-CoV-2 variants and the ancestral virus.^2^ This is explained by the fact that vaccination-induced antibodies have reduced reactivity with SARS-CoV-2 Omicron sub-lineages. In contrast, CD4 and CD8 T-cell responses do cross-react with emerging variants.^9^ Compared to the mRNA-based vaccines, primary Ad26.COV2.S vaccination yielded lower levels of S-specific antibodies, but these antibody levels remained stable for at least 6 months.^8^^,^^10^ Since S-specific neutralizing antibodies were originally identified as a correlate of protection against COVID-19^8^^,^^11^^,^^12^ booster vaccinations of Ad26.COV2.S-primed individuals were recommended to increase protection against emerging variants. Boosting Ad26.COV-2.S-primed individuals with Ad26.COV2.S, BNT162b2, or mRNA-1273 proved safe and effective,^10^^,^^13^^,^^14^ and SARS-CoV-2-specific antibody and T-cell responses are higher after heterologous boosting with an mRNA-based vaccine.^15^

SARS-CoV-2 neutralization by antibodies is predominantly dependent on targeting the receptor binding domain (RBD) or N-terminal domain (NTD) of the S protein.^16^ Mutations in these regions can lead to escape, therefore the cross-neutralization of the recently emerged Omicron sub-lineage is reduced or even absent in individuals who completed their primary regimen with any COVID-19 vaccine.^8^^,^^17^^,^^18^^,^^19^^,^^20^^,^^21^^,^^22^^,^^23^^,^^24^ However, in addition to neutralization, S-specific antibodies can have effector functions by activating cellular receptors through their constant (Fc) portion. These Fc-mediated antibody functions, like antibody-dependent cellular cytotoxicity (ADCC) and antibody-dependent cellular phagocytosis (ADCP), have been associated with reduced COVID-19 severity and mortality.^25^^,^^26^ Notably, ADCC-mediating antibodies were identified as a correlate of protection against other respiratory viral infections such as respiratory syncytial virus (RSV), influenza virus and human immunodeficiency virus (HIV)^27^^,^^28^^,^^29^ Since non-neutralizing antibodies can potentially bind epitopes spanning the entire SARS-CoV-2 S protein, including more conserved regions in the S2 domain, they could mediate broader cross-reactivity with emerging SARS-CoV-2 variants.^30^^,^^31^^,^^32^^,^^33^^,^^34^ However, considering the high number of mutations in the S protein of the Omicron sub-lineage, it is important to assess the cross-reactive capacity of antibodies that trigger non-neutralizing functions.

In addition to SARS-CoV-2-specific antibody responses, virus-specific CD4 and CD8 T cells play an important role in controlling SARS-CoV-2 infection,^35^^,^^36^^,^^37^ mainly by clearing virus-infected cells and thereby limiting disease severity.^11^ Adenovirus and mRNA-based vaccines, including Ad26.COV2.S, were shown to induce virus-specific CD4 and CD8 T cells^6^^,^^8^^,^^38^^,^^39^ that remained stable in magnitude and functionality over time. Thus far, these T-cells retained cross-reactivity with variants, including the Omicron BA.1 variant.^8^^,^^9^^,^^30^^,^^40^^,^^41^ However, how booster vaccinations in Ad26.COV2.S primed individuals affect the magnitude, breadth, and diversity of the T-cell response remains elusive.

Here, we performed immunological profiling of SARS-CoV-2-specific antibody and T-cell responses to ancestral SARS-CoV-2, and the Delta and Omicron BA.1 variants in health care workers (HCW) primed with Ad26.COV2.S and boosted with a homologous or heterologous mRNA-based vaccine. Immune responses were assessed pre-booster vaccination (3 months after priming), and 28 days after homologous or heterologous booster vaccination.

## Results

### Cohort description

For the characterization of SARS-CoV-2-specific immune responses before and 28 days after homologous or heterologous booster vaccination in Ad26.COV2.S primed individuals, n = 60 study participants were randomly selected based on the availability of samples from n = 434 healthcare workers (HCW) from the previously reported SWITCH trial.^10^ Of the 60 HCW included, n = 15 received a second vaccination with Ad26.COV2.S, n = 15 received mRNA-1273, n = 15 received BNT162b2, and n = 15 did not receive a second vaccination (no boost). Participants received their second vaccination ∼96 days (IQR 88-99 days) after priming with Ad26.COV2.S. The study design is shown in Figure S1and participant characteristics are summarized in Table 1. At baseline, before booster vaccination, there was no difference in binding antibody levels and T-cell responses measured in whole blood (Table 1, full dataset available in ^10^). Groups did not differ in female-to-male composition from our original study. There was a significant age difference; participants from the heterologous vaccination regimens had a mean age of 36 or 37 years for BNT162b2 and mRNA-1273 vaccination, respectively. In contrast, Ad26.COV2.S-boosted participants had a mean age of 51 years.

**Table 1:** Baseline characteristics

	Total	Ad26.COV.2.S/no boost	Ad26.COV.2.S/Ad26.COV.2.S	Ad26.COV2.S/mRNA-1273	Ad26.COV2.S/BNT162b2	p*-value*
	*N = 60*	*N = 15*	*N = 15*	*N = 15*	*N = 15*	
**Demographic data**						

**Sex**						

Male	20 (33)	5 (33)	6 (40)	2 (13)	7 (47)	0.25
Female	40 (67)	10 (67)	9 (60)	13 (87)	8 (53)	
**Age**	41.5 [31.8–51.0]	51.0 [38.5–55.5]	51.0 [38.5–55.0]	37.0 [28.5–43.5]	36.0 [31.0–40.0]	0.007
**BMI**	24.1 [21.1–26.6]	24.2 [21.7–26.6]	23.3 [20.8–24.6]	21.5 [20.6–25.4]	25.9 [22.0–27.3]	0.35
**Interval SV0 and SV1**	95.5 [87.5–98.8]	98.4 [88.0–99.5]	95.6 [89.5–97.9]	90.4 [87.0–98.0]	94.5 [81.0–98.5]	0.54
**Interval SV1 and SV2**	27.5 [27.4–27.6]	27.6 [27.5–27.6]	27.5 [27.5–27.6]	27.5 [27.5–27.8]	27.5 [27.5–28.1]	0.93

**Immunogenicity data**						

**Liaison**						

SV1	111.0 [54.1–212.0]	178.0 [50.6–375.5]	103.0 [71.1–194.0]	91.4 [61.5–140.5]	147.0 [54.9–309.5]	0.82
SV2	1270.0 [289.8–2962.5]	200.0 [44.5–333.0]	466 [280.5–720.0]	5050.0 [2545.0–7360.0]	2680.0 [1640.0–3965.0]	<0.001
**IGRA**						
SV1	0.24 [0.07–0.75]	0.16 [0.05–0.52]	0.18 [0.07–0.35]	0.49 [0.05–1.36]	0.38 [0.19–0.98]	0.29
SV2	0.64 [0.13–1.58]	0.09 [0.02–0.29]	0.26 [0.13–0.56]	1.43 [0.87–2.73]	1.03 [0.85–2.42]	<0.001

**Note:** Values are the number (percentage) for categorical variables and median [interquartile range] for continuous variables. SV0, study visit 0 (first vaccination); SV1, study visit 1 (prior to second vaccination); SV2, study visit 2 (potential second vaccination).

Fisher exact test was used to test differences in categorical variables.

Kruskal-Wallis test was used to test differences in continuous variables.

### Binding antibodies cross-react with the Delta and Omicron BA.1 variant

**Figure 1:**
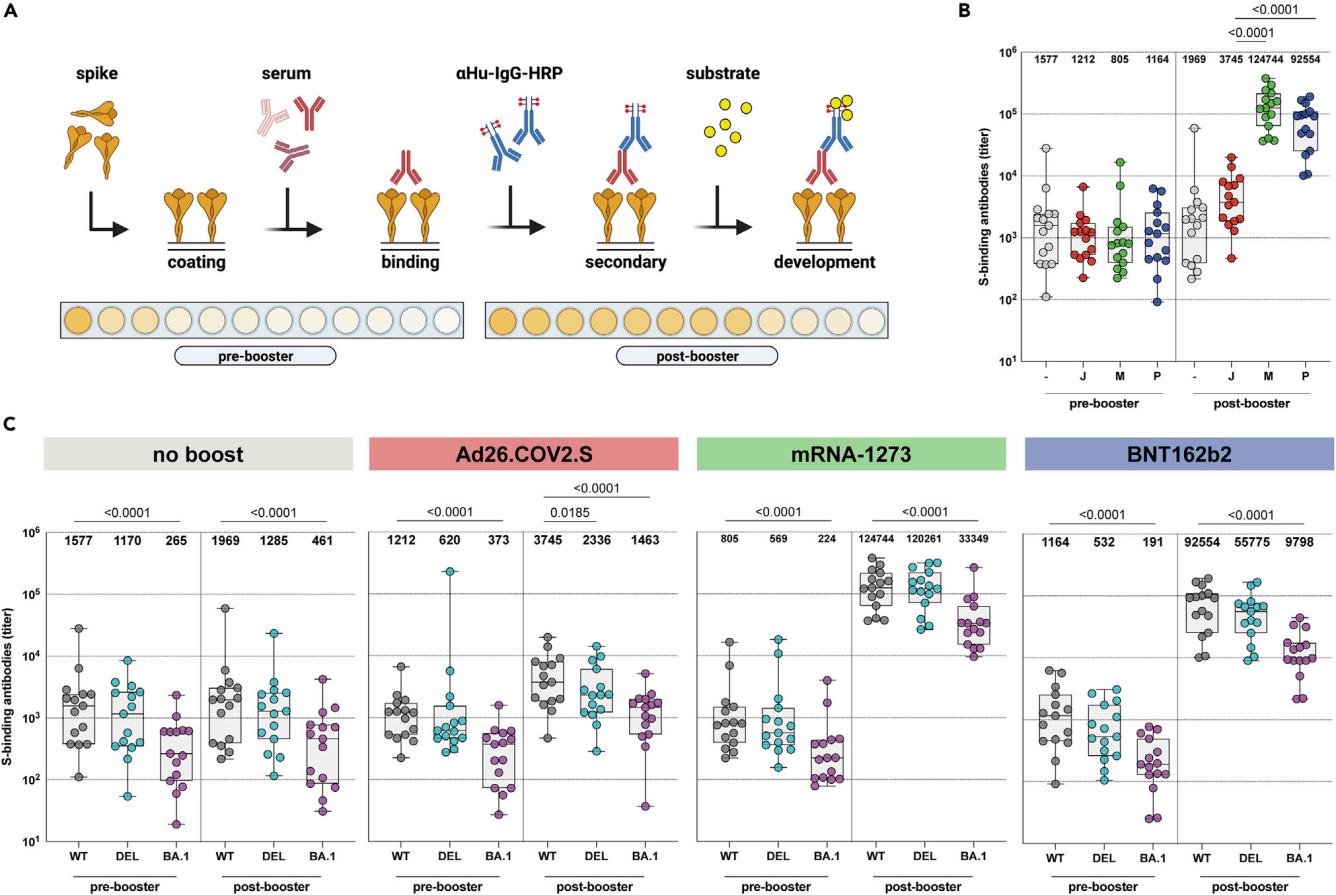
Binding antibodies are boosted by homologous or heterologous vaccination, but bind less to the Omicron BA.1 variant (A) Enzyme-linked immunosorbent assay (ELISA) methodology. (B) Binding antibodies pre- and post-booster vaccination after no boost (grey), Ad26.COV2.S boost (red), mRNA-1273 boost (green), or BNT162b2 boost (blue). Geometric mean titers (GMT) are depicted above the graph. (C) Binding antibodies against ancestral SARS-CoV-2 (grey), Delta (cyan), or Omicron BA.1 (pink) variants pre- and post-booster. GMT are depicted for each group. S = spike protein, - = no boost, J = Ad26.COV2.S, M = mRNA-1273, P = BNT162b2, WT = ancestral virus, delta = Delta variant, BA.1 = Omicron BA.1 variant. Symbols represent individual donors (n = 15 per group). Box plot depicts the median with range (min to max). Kruskal-Wallis test followed by Dunn’s multiple comparisons was performed for comparison of vaccine responses between groups; only differences between Ad26.COV2.S and mRNA-1273, or Ad26.COV2.S and BNT162b2 are shown in the figure (if a significant difference was detected). Friedman test followed by Dunn’s multiple comparisons was used to compare vaccine responses to variants within each group; only differences between ancestral SARS-CoV-2 and variants are shown in the figure (if a significant difference was detected).

Binding antibodies to ancestral SARS-CoV-2, and the Delta or Omicron BA.1 variant S proteins were assessed by ELISA (Figure 1A). A significant increase in binding antibody levels was observed 28 days after both homologous and heterologous booster vaccination (Figures 1B, S2, and S3A). We found the lowest binding antibody titer in the no-boost group (GMT of 1192). The binding antibody titers were higher after homologous (Ad26.COV2.S; GMT of 3774) and particularly after heterologous booster with mRNA-1273 (GMT of 117660) or BNT162b2 (GMT of 58747) (Figure 1B). These patterns were compared with previously reported S1-specific binding antibodies as measured by commercial assay.^10^ We found that binding antibodies were in general cross-reactive with both the Delta and Omicron BA.1 variant S proteins, although significantly lower antibody titers were found against Omicron BA.1 S across all groups and timepoints (Figures 1C and S3B). No significant differences were observed between the ancestral S protein and Delta variant. To further analyze these responses at the cellular level, we determined the percentage of total RBD-specific B cells in peripheral blood mononuclear cells (PBMC) by flow cytometry. Ancestral RBD-specific B cells were detected in the pre-booster samples of all participants and no differences were observed at baseline between the groups. Interestingly, booster vaccination did not increase the frequency, nor did it change the phenotype, of RBD-specific B cells. Similar frequencies of RBD-specific B cells, RBD-specific memory B cells as well as RBD-specific IgG memory B cells were observed pre- and post-booster with all vaccination regimens (Figure S4).

### Antibodies with Fc-mediated functions cross-react with the Delta and Omicron BA.1 variant

**Figure 2:**
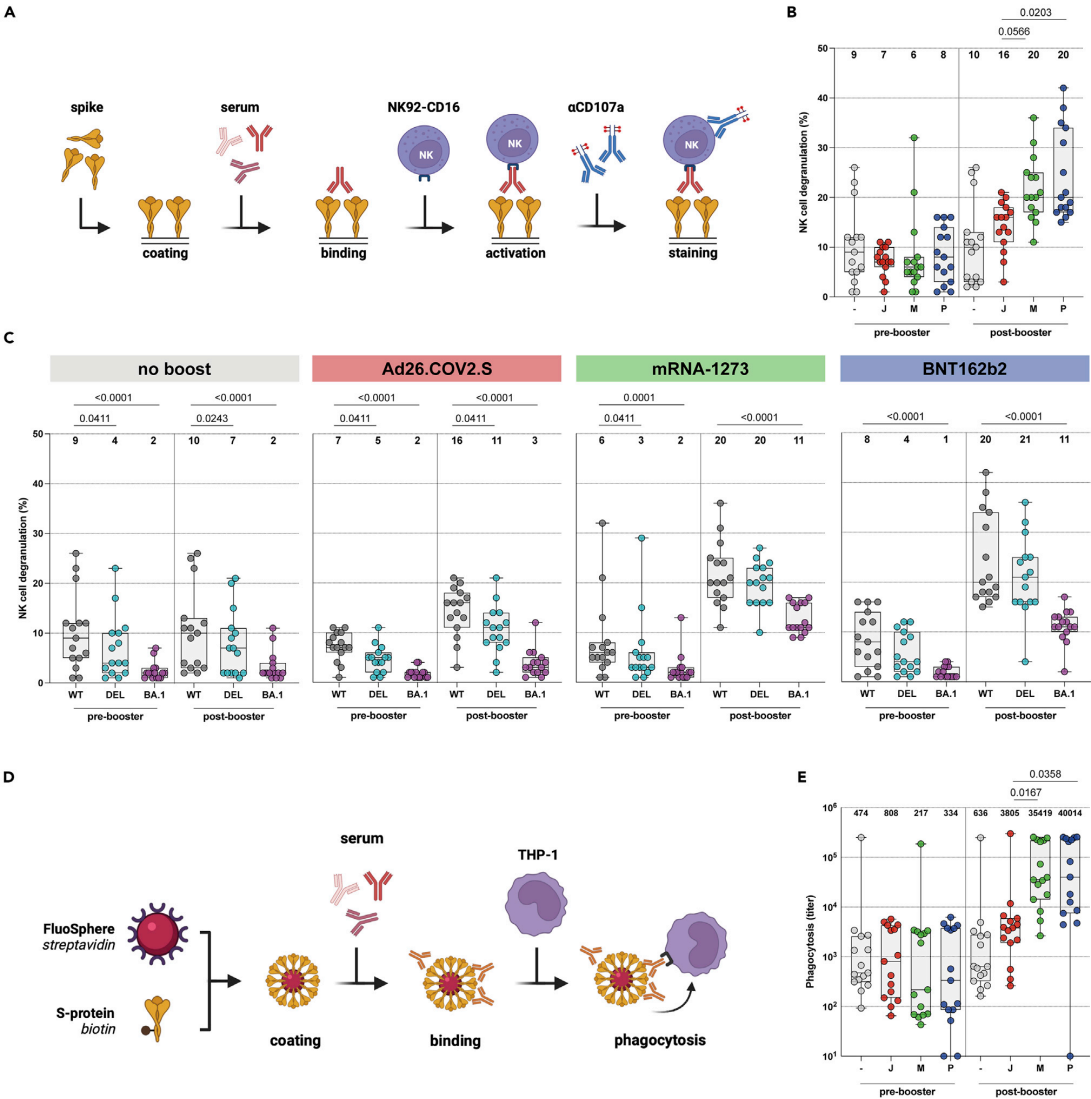
Fc-mediated antibody functions are boosted by homologous or heterologous vaccination, but less functional against the Omicron BA.1 variant (A) Antibody-dependent cell-mediated cytotoxicity (ADCC) assay methodology. (B) NK cell degranulation (%) to the ancestral SARS-CoV-2 pre- and post-booster vaccination after no boost (grey), Ad26.COV2.S boost (red), mRNA-1273 boost (green), or BNT162b2 boost (blue). Median percentages are depicted above graph. (C) NK cell degranulation to ancestral SARS-CoV-2 (grey), Delta (cyan), or Omicron BA.1 (pink) variants pre- and post-booster vaccination. Median percentages are depicted above graph. (D) Antibody-dependent cell-mediated phagocytosis (ADCP) assay methodology. (E) Phagocytosis-mediating antibodies to ancestral SARS-CoV-2 pre- and post-booster vaccination. Geometric mean titers (GMT) are depicted above graph. - = no boost, J = Ad26.COV2.S, M = mRNA-1273, P = BNT162b2, WT = ancestral virus, delta = Delta variant, BA.1 = Omicron BA.1 variant. Symbols represent individual donors (n = 15 per group). Box plot depicts the median with range (min to max). Kruskal-Wallis test followed by Dunn’s multiple comparisons was performed for comparison of vaccine responses between groups; only differences between Ad26.COV2.S and mRNA-1273, or Ad26.COV2.S and BNT162b2 are shown in the figure (if a significant difference was detected). Friedman test followed by Dunn’s multiple comparisons was used to compare vaccine responses to variants within each group; only differences between ancestral SARS-CoV-2 and variants are shown in the figure (if a significant difference was detected).

Two different Fc-mediated antibody effector functions were assessed: ADCC and ADCP. ADCC-mediating antibodies were measured in a functional NK cell degranulation assay performed on S protein-coated plates (Figure 2A). Similar to the binding antibodies, higher levels of ADCC-mediating antibodies were observed after Ad26.COV2.S booster vaccination (median of 16% degranulating cells) compared to no boost (median of 9.5%). The highest levels of ADCC-mediating antibodies were observed after mRNA-1273 (median 20%) or BNT162b2 (median of 20%) booster vaccination (Figures 2B and S5A). Although ADCC-mediating antibodies cross-reactive with the Delta variant S protein were detected in all groups at all timepoints, these were significantly lower compared to antibodies against the ancestral S protein (Figures 2C and S5B). In contrast to what was observed with binding antibodies, ADCC-mediating antibodies cross-reactive with the Omicron BA.1 S protein were only detected after mRNA-1273 (median of 11%) or BNT162b2 (median of 11%) booster vaccination (Figures 2C and S5B). Additionally, we measured ADCP-mediating antibodies in a functional THP-1 phagocytosis assay with ancestral S protein-coated beads (Figure 2D). Similarly to ADCC-mediating antibodies, Fc-mediated phagocytosis was boosted by both homologous or heterologous vaccination and highest after mRNA-1273 (GMT of 41438) or BNT162b2 (GMT of 45788) booster vaccination as compared to Ad26.COV2.S (GMT of 3373) vaccination (Figure 2E). Flow cytometric analyses and individual dilution series per vaccination regimen are shown in Figures S6A and S6B, respectively.

### Cross-neutralization of omicron BA.1 is increased after heterologous booster

**Figure 3:**
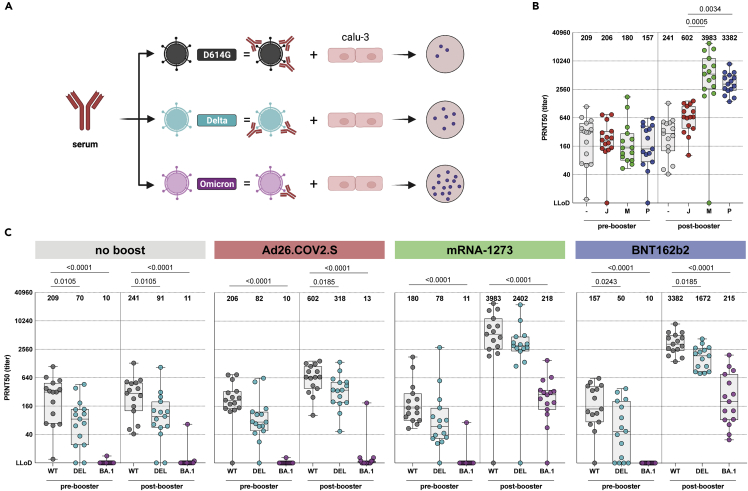
Neutralizing antibodies are boosted by homologous or heterologous vaccination, and cross-neutralize the Omicron BA.1 variant (A) Plaque-reduction neutralization test (PRNT) assay methodology. (B) PRNT50 titer to ancestral SARS-CoV-2 pre- and post-booster vaccination after no boost (grey), Ad26.COV2.S boost (red), mRNA-1273 boost (green), or BNT162b2 boost (blue). Geometric mean titers (GMT) are depicted above graph. (C) PRNT50 titer pre- and post-booster vaccination for ancestral SARS-CoV-2 (grey), Delta (cyan), or Omicron BA.1 (pink) variants. Geometric mean titers (GMT) are depicted above graph. PRNT50 = plaque reduction neutralization test 50% end-point, - = no boost, J = Ad26.COV2.S, M = mRNA-1273, P = BNT162b2, WT = ancestral virus, delta = Delta variant, BA.1 = Omicron BA.1 variant. Symbols represent individual donors (n = 15 per group). Box plot depicts the median with range (min to max). Kruskal-Wallis test followed by Dunn’s multiple comparisons was performed for comparison of vaccine responses between groups; only differences between Ad26.COV2.S and mRNA-1273, or Ad26.COV2.S and BNT162b2 are shown in the figure (if a significant difference was detected). Friedman test followed by Dunn’s multiple comparisons was used to compare vaccine responses to variants within each group; only differences between ancestral SARS-CoV-2 and variants are shown in the figure (if a significant difference was detected).

Neutralizing antibodies were assessed in an infectious virus neutralization assay with the ancestral SARS-CoV-2, and the Delta, and Omicron BA.1 variants (Figure 3A). mRNA-based booster vaccination after Ad26.COV2.S priming led to the highest levels of neutralizing antibodies against the ancestral SARS-CoV-2, GMT of 3983 and GMT 3382, respectively (Figures 3B and S7A). Cross-neutralizing antibodies against Delta and Omicron BA.1 were observed after mRNA-based booster vaccination, although at a significantly lower level compared to the ancestral SARS-CoV-2. Strikingly, cross-neutralization of the Omicron BA.1 variant was virtually absent (GMT of 13) after Ad26.COV2.S booster vaccination (Figures 3C and S7B). Individual S-curves per vaccination regimen are shown in Figure S8.

### Correlations between serological assays

**Figure 4:**
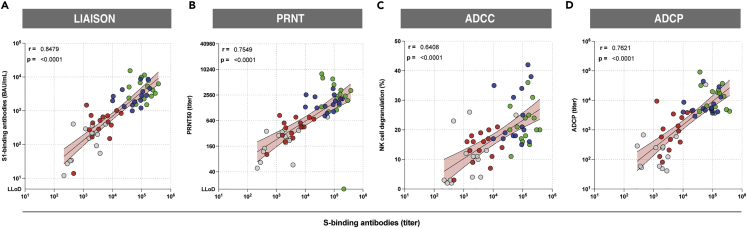
Functional antibody responses correlate with antibody binding to the ancestral SARS-CoV-2 S protein (A) Correlation between S1-specific binding antibodies measured by Liaison and S-specific binding antibodies measured by ELISA. (B) Correlation between neutralizing antibodies and S-specific binding antibodies measured by ELISA (C) Correlation between NK cell degranulation mediating S-specific antibodies (ADCC) and S-specific antibody binding measured by ELISA. (D) Correlation between phagocytosis-mediating antibody titers (ADCP) and S-specific binding antibodies as measured by ELISA. Colors represent different booster groups: no boost (grey), Ad26.COV2.S boost (red), mRNA-1273 boost (green), and BNT162b2 boost (blue). Symbols represent individual donors post-booster vaccination (n = 15 per group). Simple linear regression analysis on log-transformed data was used to calculate Spearman’s correlation coefficient and p-values.

We examined the correlations between S-specific binding antibodies and S1-binding antibodies (Figure 4A), and their functionalities including neutralization (PRNT50) (Figure 4B), NK cell degranulation (ADCC) (Figure 4C), and phagocytosis (ADCP) (Figure 4D) against the ancestral SARS-CoV-2 and found all correlations to be positive and significant (p < 0.05). We additionally performed correlations for the ancestral-, Delta- and BA.1-specific responses per assay (Figure S9). We observed a direct relationship between ancestral- and variant-specific antibody levels and found that reduced (or absent) variant-specific antibody responses were directly related to low total antibody levels.

### S-specific CD4 and CD8 T cells cross-react with Delta and Omicron BA.1

**Figure 5:**
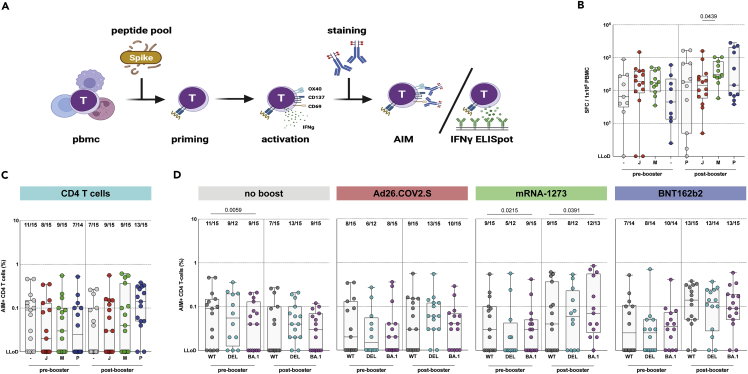
SARS-CoV-2-specific CD4 T cells cross-react with Omicron BA.1 (A) Activation-induced marker (AIM) assay and IFN-ɣ ELISPOT methodology. (B) IFN-ɣ secreting T cells after stimulation with an overlapping S peptide pool from the ancestral SARS-CoV-2 pre- and post-booster vaccination, after no boost (grey, n = 9), Ad26.COV2.S boost (red, n = 14), mRNA-1273 boost (green, n = 11), or BNT162b2 boost (blue, n = 11). (C) Comparison of CD4 T-cell responses to ancestral SARS-CoV-2. (D) CD4 T-cell responses against ancestral SARS-CoV-2 (grey), Delta (cyan), or Omicron BA.1 (pink) variants pre- and post-booster vaccination. Fraction of donors with measurable AIM-positive T-cells is indicated above the box-plots. - = no boost, J = Ad26.COV2.S, M = mRNA-1273, P = BNT162b2, WT = ancestral virus, delta = Delta variant, BA.1 = Omicron BA.1 variant. Symbols represent individual donors. Box plot depicts the median with range (min to max). Kruskal-Wallis test followed by Dunn’s multiple comparisons was performed for IFN-ɣ ELISPOT. Mann-Whitney U test was performed for comparison of vaccine groups Ad26.COV2.S and mRNA-1273, and Ad26.COV2.S and BNT162b2 (shown in panel C); a p-value of 0.025 was considered significant after Bonferroni correction. Wilcoxon rank test was performed for the comparison of variant-specific T-cell responses between ancestral SARS-CoV-2 and variants (shown in panel D); a p-value of 0.025 was considered significant after Bonferroni correction.

Next, we measured T-cell responses before and after homologous or heterologous booster vaccination. To directly assess T-cell responses in whole blood, we previously performed an interferon gamma (IFNγ) release assay (IGRA), and found that T-cell responses were boosted by both homologous and heterologous booster vaccination.^10^ To assess T-cell responses in depth, PBMCs were stimulated with overlapping peptide pools spanning the full-length ancestral S protein, and responses were measured via IFN-γ ELISPOT (Figure 5A). Here, we found that mRNA-1273 booster vaccination induced significantly higher numbers of IFN-γ producing T cells to ancestral SARS-CoV-2 compared to homologous booster vaccination (Figure 5B).

To measure variant-specific responses, PBMCs were stimulated with overlapping peptide pools representing the full-length S protein from the ancestral SARS-CoV-2, and the Delta and Omicron BA.1 variants (Figure 5A). Following stimulation, CD4 (OX40^+^CD137^+^) and CD8 (CD69^+^CD137^+^) T cell activation-induced marker (AIM) expression was measured by flow cytometry (Figure S10A). CD4 and CD8 T-cell responses were detected in 32/60 (53%) of participants pre-booster, and levels were comparable between groups. Booster vaccination with either Ad26.COV2.S or mRNA-1273 did not significantly increase CD4 T-cell responses. Interestingly, booster vaccination with BNT162b2 increased the number of participants with a measurable CD4 T-cell response from 7/14 (50%) to 13/15 (87%), with a significantly higher percentage of activated CD4 T cells (GM of 0.03% to 0.1%) after booster vaccination. In contrast, CD4 T-cell responses waned for the no boost group (11/15 responders at baseline to 7/15 responders 28 days later) (Figures 5C and S10B). CD4 T-cell reactivity with the Delta and Omicron BA.1 variant was maintained, and comparable to reactivity with the ancestral S protein (Figures 5D and S10B). As for CD8 T-cell responses, no clear boosting effect of either homologous or heterologous vaccination was observed on basis of the number of responders (Figure S10C). Similar to CD4 T cells, CD8 T cells equally reacted with all SARS-CoV-2 variants tested (Figure S10C).

### mRNA-based booster vaccination led to the expansion of S-specific T-cell clones

**Figure 6:**
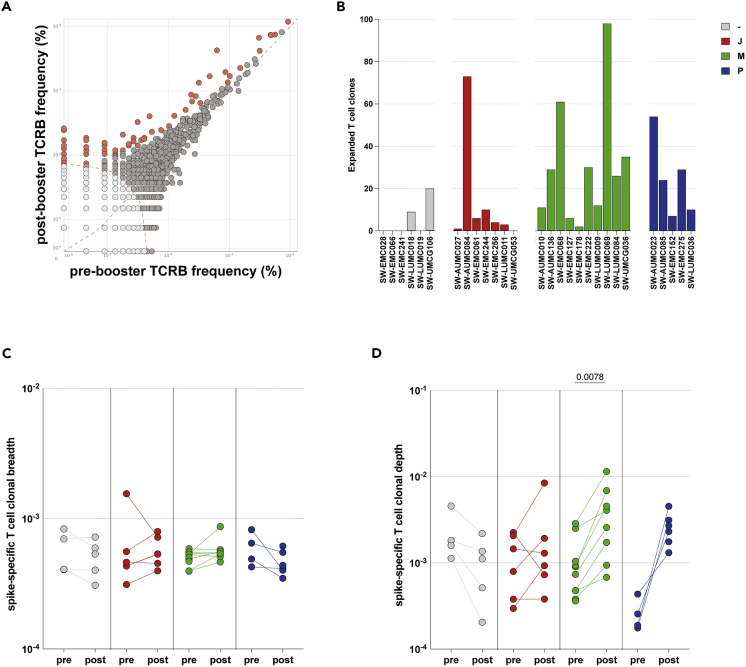
Expansion, breadth, and depth of SARS-CoV-2-specific T-cell response (A) Representative analysis of clone expansion based on the amino acid sequence of the TCR pre- (T1) and post-booster (T2) vaccination. Grey symbols represent individual T-cell clones that did not significantly expand upon booster vaccination. Orange symbols represent those T-cell clones that expanded following booster vaccination. Symbols on the X- or Y-axis are unique clones for the respective time-point. (B) Expanded SARS-CoV-2 S-specific T-cell clones following vaccination with no boost (grey), Ad26.COV2.S boost (red), mRNA-1273 boost (green), or BNT162b2 boost (blue). (C and D) Breadth and (D) depth of the T-cell response pre- and post-booster vaccination. - = no boost (n = 4 pre-boost/n = 5 post-boost), J = Ad26.COV2.S (n = 6), M = mRNA-1273 (n = 10 pre-boost/n = 8 post-boost), P = BNT162b2 (n = 4 pre-boost/n = 6 post-boost). Symbols represent individual donors. Box plot depicts the median with range (min to max). Wilcoxon rank test was performed for the comparison of T-cell breadth and depth pre- and post-booster vaccination.

We further evaluated the expansion, breadth, and depth of the SARS-CoV-2-specific T-cell response after different booster regimens. TCRβ sequencing was performed to define the repertoires of N = 30 participants (N = 7 no boost, N = 7 Ad26.COV2.S boost, N = 10 mRNA-1273 boost, and N = 6 BNT162b2 boost) pre- and post-booster vaccination.^42^ Initially, we compared clones pre- and post-booster vaccination within donors to identify expanding clones after booster vaccination (representative example shown in Figure 6A). Expanding clones were detected in two donors that did not receive a boost, but in a time period of 28 day background expansion of ±5-10 clones can be expected (Figure 6B). More expanding clones were observed in the Ad26.COV2.S-boosted individuals as compared to no boost (dominated by 73 expanding clones in 1 individual), but especially in the mRNA-1273 and BNT162b2-boosted individuals the number of expanding clones was often >20 (Figure 6B).

To identify SARS-CoV-2-specific T-cell clones, the TCR sequences were compared to a sequence dataset (the ImmunoCODE MIRA dataset) enriched in COVID-19 cases versus controls.^43^ This method identifies clones that are specific to SARS-CoV-2 and reduces noise associated with clones that are very frequent or potentially cross-reactive. Breadth (number of unique SARS-CoV-2-specific TCRs) and depth (frequency of SARS-CoV-2-specific TCRs) were calculated for S- and ORF1ab-, ORF3a-, M− and N-specific T cells. As expected, a dominant S-specific T-cell response was detected, as SARS-CoV-2-infected donors were excluded from this study (Figures S11A and S11B). Interestingly, booster vaccinations did not lead to a significant increase in the breadth of the S-specific T-cell response (Figure 6C). However, booster vaccination with mRNA-1273 led to a significant increase in the depth/frequency (Figure 6D) of the SARS-CoV-2-specific T-cell response, which was not observed after Ad26.COV2.S booster vaccination.

## Discussion

We performed immunological profiling of the SARS-CoV-2-specific immune response, including reactivity to the Delta and Omicron BA.1 variants, after homologous or heterologous booster vaccination of Ad26.COV2.S-primed individuals. We found that Ad26.COV2.S priming provided a solid immunological base for strong and broad SARS-CoV-2-specific immune responses upon subsequent mRNA-based booster vaccination. A limitation of this study was that the age was significantly different between the groups that received an mRNA-based booster vaccination, Ad26.COV2.S boost, or no boost. This potentially contributed to the fact that we concluded that mRNA-based vaccines are superior boosters, as older age was reported as a negative factor contributing to the induction of lower antibody titers following SARS-CoV-2 infection.^44^ However, in the original SWITCH trial, with a larger number of participants, we also found the superior boosting capacity of mRNA-based vaccines, despite this difference.^10^ Additionally, other studies reported similar superiority of mRNA-based over vector-based COVID-19 vaccines.^45^^,^^46^ Samples were collected between August and September of 2021 when Omicron sub-lineages were not circulating in the Netherlands. To exclude recent infections, a nucleocapsid (N) ELISA was performed on all samples before participants received their booster vaccination.^10^

Here, we compared four different booster regimens in a random selection of individuals from the larger SWITCH study.^10^ Binding antibodies targeting the ancestral SARS-CoV-2, and the Delta and Omicron BA.1 variants, increased after booster vaccination and levels were highest in participants that received an mRNA-based booster. Strikingly, we found that the proportion of RBD-specific memory B cells in blood did not increase after booster vaccination. This indicates that the original Ad26.COV2.S priming induced a sustained RBD-specific memory B cell response and we speculate that final maturation had already occurred in the 3 months after the initial vaccination. This is in line with the slow increase of S1-specific antibodies after priming vaccination with Ad26.COV2.S and the stable levels of these antibodies.^8^ Therefore, booster vaccination led to the rapid induction of antibody production by memory B cells rather than expansion of SARS-CoV-2-specific memory B cells. As SARS-CoV-2-specific B cells were measured in peripheral blood, it cannot be excluded that booster vaccination resulted in the expansion of the memory B cells in lymphoid tissues.

Antibodies can have a multitude of effector functions, ranging from direct neutralization to Fc-mediated triggering of cytotoxicity or phagocytosis targeting infected cells and/or cell-free virions, depending on the antibody isotype, glycosylation pattern, and Fc receptor bound.^47^ The majority of the participants in this study developed neutralizing antibodies against the ancestral SARS-CoV-2 (independent of the vaccination regimen). Neutralizing antibodies targeting the Delta variant were readily detected at slightly lower levels, but neutralizing antibodies targeting Omicron BA.1 could only be detected after mRNA-based booster vaccination, at considerably lower levels compared to the ancestral SARS-CoV-2.^8^ Importantly, mRNA-based booster vaccination resulted in significantly higher neutralizing antibody titers as compared to Ad26.COV2.S booster vaccination.

We assessed Fc-mediated effector functions of antibodies. It was previously hypothesized that these functions might play a role in contributing to protection against COVID-19,^48^^,^^49^ but relatively little is known about the impact of Fc-mediated antibody effector functions.^34^ Novel antigenically distinct SARS-CoV-2 variants, like the Delta variant and Omicron sub-lineages, are partly capable of evading neutralizing antibodies by accumulating mutations in the RBD.^1^^,^^50^^,^^51^^,^^52^ Functional non-neutralizing antibodies are speculated to be less susceptible to immune escape by emerging variants, as they are not dependent on the recognition of specific epitopes in the RBD and they can target the entire S protein.^25^^,^^34^ Here, we show an increase in ADCC- and ADCP-mediating antibodies against the ancestral SARS-CoV-2, Delta, and Omicron BA.1 variants following both homologous and heterologous booster vaccination. Similar to the neutralizing antibody responses, Fc-mediated antibodies were higher following mRNA-based booster vaccination. Although effector functions mediated by non-neutralizing antibodies were also reduced towards the Delta and Omicron BA.1 variant, ADCC-mediating antibodies were still clearly detected after mRNA-based booster vaccination. We speculate that escape from antibodies with the potential to target the entire S protein is caused by the numerous mutations in Omicron BA.1, even outside the RBD (and NTD). For ADCP we were not able to measure variant-specific responses due to a lack of the required reagents. However, based on the observed correlation between binding, ADCC-mediating, and ADCP-mediating antibodies, we expect similar patterns of cross-reactivity.

SARS-CoV-2-specific T cells play an important role in reducing COVID-19 severity following re- or breakthrough infection.^53^ T cells can clear virus-infected cells, contributing to the reduction of virus replication.^11^ Virus-specific T cells are thought to be long-lived, as these have been detected up to six months after the completion of primary vaccination regimens,^8^ and up to 17 years after SARS-CoV infection.^36^ T cells can target epitopes dispersed throughout proteins, including conserved epitopes under functional constraints, and therefore retain cross-reactivity to SARS-CoV-2 variants,^9^^,^^30^^,^^40^^,^^41^ including the Omicron sub-lineage.^7^^,^^8^^,^^54^ Here, we show that T-cell responses are boosted in Ad26.COV2.S-primed individuals especially after mRNA-based booster vaccination, as measured by both IFN-γ levels and expansion of S-specific T-cell clones. However, these T-cell responses were not significantly higher than after Ad26.COV2.S homologous booster vaccination. Alternatively, the contraction phase after mRNA-based boost is reported to be rapid,^55^ leaving a small window of opportunity to detect virus-specific T-cell increases after booster vaccination. Although based on TCRβ sequencing the breadth of the s-specific response did not increase after heterologous booster vaccination, reactivity of both CD4 and CD8 T cells with the Delta and Omicron BA.1 variants was retained. No significant increase in CD8 T-cell responses was detected following any of the booster vaccinations, following the same pattern as CD4 T-cell responses. However, high variability in the AIM results and less sensitivity in detecting SARS-CoV-2-specific CD8^+^ T-cells makes it more complicated to interpret these data.

Currently, several sub-lineages of the Omicron variant are circulating. Although the BA.1 lineage quickly became dominant upon introduction, it was rapidly replaced by the BA.2 lineage. Both variants have shown significant escape from neutralizing antibodies.^17^^,^^20^^,^^56^^,^^57^ Currently, other Omicron variants are rapidly establishing dominance in different geographical locations,^21^^,^^22^^,^^23^ and escape has been demonstrated for the newer Omicron variants.^24^ In our study, we have focused on cross-reactive immune responses to Omicron BA.1, since at the time of the experiments the newer variants were not yet circulating. Based on cross-reactivity with BA.1 and available literature, we expect that non-neutralizing antibodies and T-cell responses have at least equal potential for cross-reactivity with these novel immune-evasive variants, based on the targeting of conserved epitopes.

In conclusion, we showed that Ad26.COV2.S priming provided a solid immunological base for SARS-CoV-2-specific immune responses triggered by mRNA-based booster vaccination. Additionally, we show that heterologous mRNA-based boosters are more potent compared to homologous Ad26.COV2.S boosting. Neutralizing antibodies targeting immune-evasive variants were detectable after a mRNA-based booster, and non-neutralizing antibodies and T-cell responses to these variants were retained or even boosted. These findings are similar to previous findings in individuals primed with another vector-based vaccine (ChAdOx1-S), who received an mRNA-based booster vaccination.^46^ It is crucial to further investigate how these responses to booster vaccination compare to individuals primed with ChAdOx1-S or an mRNA-based vaccine, and whether the initial priming vaccination still has an effect on ongoing booster campaigns with bivalent vaccines. Although there currently is a high prevalence of breakthrough infections with viruses from the newly emerging Omicron sub-lineages, the related disease has been reported to be relatively mild.^58^ Non-neutralizing antibodies and memory T cells are expected to play an important role in reducing COVID-19 disease severity and boosting these could be crucial for vaccine effectiveness in the future.^53^^,^^59^

### Limitations of the study

Because of the complexity of the used techniques, and the amount of different immunological parameters studied, it was not feasible to test large cohorts in a high-throughput setting. Therefore, we present a relatively small dataset. Although most immunological parameters show a clear pattern, the TCR-sequencing data are limited by the small dataset and should be interpreted carefully. We expanded the analysis of functional antibodies beyond neutralization and included experiments to measure Fc-mediated effector functions. We could not analyze all Fc-mediated effector functions against the variants of interest, as not all reagents were available at the time of the study. Additionally, we focused our analyses around cross-reactivity of immune responses with Omicron BA.1, which was dominant at the time experiments were performed. Upcoming experiments will include circulating variants like BA.5, BQ.1.1, and XBB.

## Consortia

This study was published on behalf of the SWITCH-ON consortium, including: Nathalie Tjon, Karenin van Grafhorst, Leanne P.*M*. *van* Leeuwen, Faye de Wilt, Sandra Scherbeijn, Aldert C.P. Lamoré, Hannah M. Garcia Garrido, Agnes M. Harskamp, Irma Maurer, Arginell F. Girigorie, Brigitte D. Boeser-Nunnink, Marga M. Mangas Ruiz, Karel *A*. *van* Dort, Jacqueline J. de Vries-Idema, Jopie Zuidema, Jessica A. Vlot, Petra H. Verbeek –Menken, Annelies Van Wengen-Stevenhagen.

## STAR★Methods

### Key resources table

**Table:** 

REAGENT or RESOURCE	SOURCE	IDENTIFIER
**Antibodies**		

Anti-human CD3 PerCP clone SK7	BD Biosciences	Cat#345766; RRID:AB_2783791
Anti-human CD4 V450 clone L200	BD Biosciences	Cat#560811; RRID:AB_2033927
Anti-human CD8 FITC clone DK25	Agilent	Cat#F076501-2; RRID:AB_578668
Anti-human CCR7 BV711 clone 150503	BD Biosciences	Cat#566602; RRID:AB_2739758
Anti-human CD45RA PECy7 clone L48	BD Biosciences	Cat#337186; RRID:AB_2828012
Anti-human CD69 APC-H7 clone FN50	BD Biosciences	Cat#560737; RRID:AB_1727508
Anti-human CD137 PE clone 4B4-1	Miltenyi Biotec	Cat#130-119-885; RRID:AB_2783944
Anti-human OX40 BV605 clone L106	BD Biosciences	Cat#745217; RRID:AB_2742808
LIVE/DEAD Fixable Aqua Dead Cell staining AmCyan	Invitrogen	Cat#L34957
Anti-human CD56 PE clone B159	BD Biosciences	Cat#555516; RRID:AB_395906
Anti-human CD107a V450 clone H4A3	BD Biosciences	Cat#561345; RRID:AB_10646032
Rabbit polyclonal anti-human IgG HRP	Dako	Cat#P021402-2
Goat anti-rabbit IgG HRP	Dako	Cat#P044801-2
Anti-human IFN-γ clone 1-D1K	Mabtech	Cat#3420-3-1000; RRID:AB_907282
Anti-human biotinylated IFN-γ clone 7-B6-1	Mabtech	Cat#3420-6-1000; RRID:AB_907272

**Bacterial and virus strains**		

SARS-CoV-2 D614G primary isolate	This study	GISAID: hCoV-18/Netherlands/ZH-EMC-2498
SARS-CoV-2 Delta primary isolate	This study	GISAID: hCoV-19/Netherlands/NB-MVD-CWGS2201159
SARS-CoV-2 BA.1 primary isolate	This study	GISAID: hCoV-19/Netherlands/LI-SQD-01032/2022

**Biological samples**		

Plasma	This study	N/A
PBMC	This study	N/A
Nanogram 100 mg/mL solution for infusion	Sanquin	Cat#RVG118226
Bovine serum albumin (BSA)	Sigma	Cat#A8327-50ML
Fetal bovine serum (FBS)	Merck Life Science	Cat#F7524-500ML
Horse serum	Merck Life Science	Cat#H1270-500ML
Human AB (hAB) serum; inactivated	Sigma	Cat#H6914

**Chemicals, peptides, and recombinant proteins**		

SARS-CoV-2 Wu-Hu1 Spike Megapool	This study	N/A
SARS-CoV-2 Delta Spike Megapool	This study	N/A
SARS-CoV-2 BA.1 Spike Megapool	This study	N/A
DMSO	Honneywell	Cat#D5879-500ML
PMA	Merck Life Science	Cat#P1585-1MG
Ionomycin	Merck Life Science	Cat#I0634-5MG
Blocker blotto in TBS	Invitrogen	Cat#37530
Tween-20	Merck Life Science	Cat#P1379-1L
3,3′,5,5′-tetramethylbenzidine substrate	KPL	Cat#5120–0038
Sulfuric acid	Millipore	Cat#1.09073.1000
Lymphoprep	Stemcell Technologies	Cat#07861
Benzonase, purity grade 2	Millipore	Cat#1.01654.0001
SARS-CoV-2 biotinylated monomeric S protein D614G	Sino Biological	Cat#40589-V27B-B_100UG
SARS-CoV-2 trimeric S protein D614G	Sino Biological	Cat#40589-V08H6-100UG
SARS-CoV-2 trimeric S protein Delta	Sino Biological	Cat#40589-V08H10_100UG
SARS-CoV-2 trimeric S protein BA.1	Sino Biological	Cat#40589-V08H26_100UG
GolgiStop	BD Biosciences	Cat#554724
GolgiPlug	BD Biosciences	Cat#555029
Cytofix/Cytoperm	BD Biosciences	Cat#554722
Neutravidin FluoSphere beads	Life Technologies	Cat#F8775
Formaldehyde solution 37%	VWR	Cat#F1635-500ML
SARS-CoV-2 S1+S2 peptide pool	JPT Peptide Technologies	Cat#PM-WCPV-S
PHA	Remel Europe Ltd	Cat#HA16
Poly-HRP buffer	ThermoFisher	Cat#N500
Streptavidin poly-HRP	Sanquin	Cat#M2051
TMB substrate	Mabtech	Cat#2651–10
2-Mercaptoethanol	Merck Life Science	Cat#M3148-25ML
NaHCO3	Merck Life Science	Cat#S5761-500G
L-glutamine	Capricorn Scientific	Cat#GLN-B
Myo-inositol	Sigma	Cat#15125–50G
Folic acid	Sigma	Cat#F7876-25G
Sodium pyruvate 100mM	Gibco	Cat#11360–039
X-VIVO medium	Lonza	Cat#BE02-060F

**Critical commercial assays**		

Liaison SARS-CoV-2 TrimericS IgG assay	DiaSorin	Cat#311510
QuantiFERON SARS-CoV-2 Blood Collection Tubes	Qiagen	Cat#626725
QuantiFERON ELISA	Qiagen	Cat#626410
DNA mini kit	Qiagen	Cat#51306
SARS-CoV-2 RBD B cell analysis kit	Miltenyi Biotec	Cat#130-128-022
immunoSEQ Assay	Adaptive Biotechnologies	N/A

**Experimental models: Cell lines**		

NK92.05 – CD16	Kerry Campbell	Kind gift
THP-1	ATCC	Cat#TIB-202
Calu-3	ATCC	Cat#HTB-55

**Software and algorithms**		

FlowJo v.10.8.1	TreeStar	N/A
PRISM v.9.4.1	GraphPad	N/A

**Other**		

FACSLyric	BD Biosciences	N/A
Immunospot Imaging Analyzer	CTL Europe GmbH	N/A
ELISA microtiter plate reader Infinite F200	Tecan	N/A
FACS Canto II	BD Biosciences	N/A
SepMate-50 (IVD)	Stemcell Technologies	Cat#85460

### Resource availability

#### Lead contact

Further information and requests for resources and reagents should be directed to and will be fulfilled upon reasonable request by the lead contact, Dr. Rory D. de Vries (r.d.devries@erasmusmc.nl).

#### Materials availability

SARS-CoV-2 peptide pools used in this study are from Alessandro Sette (alex@lji.org), and are available upon reasonable request with a completed materials transfer agreement. Other unique/stable reagents generated in this study are available from the lead contact with a completed materials transfer agreement.

### Experimental model and subject details

#### NK92.05 - CD16 cell line

The NK92.05 human cell line has been genetically modified to express a high affinity CD16 fc-receptor through a mutation at 176V. NK92.05 cells were cultured in Alpha-MEM supplemented with NaHCO_3_ (2.2 g/L, pH 7.2), 2-mercapthoethanol (0.0001 M), L-glutamine (200 mM, Gibco), myo-inositol (0.2 mM), 10% horse serum, 10% fetal bovine serum, folic acid (0.004 mM), sodium pyruvate (1 mM) penicillin (100 IU/mL), and streptomycin (100 μg/mL). 100 U/mL IL-2 was added to the medium upon thawing of the cell line, which was reduced to a maintenance dose of 50 U/mL for stable culturing. Medium was refreshed twice a week and fresh IL-2 was added to the culture. For the ADCC assay, culture medium was replaced by Alpha-MEM supplemented with NaHCO_3_ (2.2 g/L, pH 7.2), L-glutamine (200 mM, Gibco), 10% fetal bovine serum, penicillin (100 IU/mL), and streptomycin (100 μg/mL).

#### THP-1 cell line

The THP-1 human cell line is a monocyte cell isolated from peripheral blood of a male monocytic leukemia patient. THP-1 cells were cultured in RPMI1640 supplemented with 10% fetal bovine serum, 2-mercapthoethanol (0.05 mM), penicillin (100 IU/mL), and streptomycin (100 μg/mL).

### Method details

#### Study design

The SWITCH trial is a single-(participant)-blinded, multi-center, randomized controlled trial among HCWs without severe comorbidities performed in four academic hospitals in the Netherlands (Amsterdam University Medical Center, Erasmus University Medical Center, Leiden University Medical Center, and University Medical Center Groningen), according to the published protocol.^60^ The trial adheres to the principles of the Declaration of Helsinki and was approved by the Medical Research Ethics Committee from Erasmus Medical Center (MEC 2021–0132) and the local review boards of participating centers. All participants provided written informed consent before enrollment.

#### Participants

For analysis of humoral and cellular immune responses, 60 donors were randomly selected, taking into account whether sufficient material was available. Participants randomly selected for immunological profiling received a priming vaccination with Ad26.COV2.S, followed by a booster vaccination with Ad26.COV2.S, mRNA-1273 or BNT162b2 after ±95 days (N = 15 per group). This differs from the complete original study group, in which the participants received their second vaccination ±84 days after priming with Ad26.COV2.S. As a control group, Ad26.COV2.S primed individuals that were not boosted were included (N = 15). Blood samples were collected at day 0 (pre-booster) and day 28 (post-booster), also for the non-boosted control group (Figure S1A).

#### PBMC and serum isolation

Blood was collected in vacutainer® SST tubes (BD), serum was obtained and stored at −20°C for further experiments. PBMC were isolated from blood and collected in vacutainer tubes containing lithium heparin as anticoagulant by density gradient centrifugation with Lymphoprep™ (Stemcell Technologies) in 50 mL SepMate™ collection tubes (Stemcell Technologies) according to manufacturer’s instructions. Briefly, blood was diluted in phosphate buffered saline (PBS), loaded onto Lymphoprep™ and PBMCs were separated by centrifugation at 2000 g for 15 minutes. PBMCs were washed 3 times in PBS, counted and frozen in 90% fetal bovine serum (FBS) with 10% DMSO (Honeywell) in liquid nitrogen.

#### Detection of S1-specific binding antibodies

Serum samples were tested for anti-S1 immunoglobulin (Ig)G antibodies using a validated Liaison SARS-CoV-2 TrimericS IgG assay (DiaSorin, Italy).^8^^,^^30^ The lower limit of detection (LLoD) was set at 4.81 binding arbitrary units (BAU)/mL and the responder cut-off at 33.8 BAU/mL. The assay was performed according to manufacturer’s instructions.

#### Virus neutralization assay (PRNT50)

Serum samples were tested for the presence of neutralizing antibodies against ancestral SARS-CoV-2, and the Delta and Omicron (BA.1) variants in a plaque reduction neutralization test (PRNT). Viruses were cultured from clinical material, sequences were confirmed by next-generation sequencing: D614G (ancestral, GISAID: hCov-19/Netherlands/ZH-EMC-2498), B.1.617.2 (Delta, GISAID: hCoV-19/Netherlands/NB-MVD-CWGS2201159/2022), and B.1.1.529 (Omicron BA.1, GISAID: hCoV-19/Netherlands/LI-SQD-01032/2022). The human airway Calu-3 cell line (ATCC HTB-55) was used to grow virus stocks and for PRNT. Calu-3 cells were cultured in OptiMEM (Gibco) supplemented with Glutamax, penicillin (100 IU/mL), streptomycin (100 IU/mL), and 10% fetal bovine serum (FBS). In short, heat-inactivated sera were diluted two-fold in OptiMEM without FBS starting at a 1:10 dilution or in the case of a S1-specific antibody level >2500 BAU/mL starting at 1:80 in 60 μL. 400 PFU of each SARS-CoV-2 variant in 60 μL OptiMEM medium was added to diluted sera and incubated at 37°C for 1 hour. Antibody-virus mix was transferred onto Calu-3 cells and incubated at 37°C for 8 hours. Cells were fixed in PFA and stained with polyclonal rabbit anti-SARS-CoV-2 nucleocapsid antibody (Sino Biological) and a secondary peroxidase-labeled goat-anti rabbit IgG antibody (Dako). Signal was developed with precipitate-forming 3,3′,5,5′-tetramethylbenzidine substrate (TrueBlue; Kirkegaard & Perry Laboratories) and the number of plaques per well was counted with an ImmunoSpot Image Analyzer (CTL Europe GmbH). The 50% reduction titer (PRNT50) was estimated by calculating the proportionate distance between two dilutions from which the endpoint titer was calculated. Infection controls (no sera) and positive serum control (Nanogram® 100 mg/mL, Sanquin) were included on each plate. A PRNT50 value one dilution step (PRNT50 = 10) lower than the lowest dilution was attributed to samples with no detectable neutralizing antibodies.

#### Enzyme-linked immunosorbent assay (ELISA)

Binding antibodies against ancestral SARS-CoV-2, and the Delta and Omicron (BA.1) variants were determined by a in-house developed ELISA.^61^ Briefly, ELISA high-binding EIA/RIA plates (Costar) were coated (20 ng/well) with baculovirus-generated trimeric prefusion His-tagged S protein from ancestral SARS-CoV-2 (D614G), and Delta and Omicron BA.1 variants (Sino Biological) at 4°C overnight. Next, plates were blocked with blocker blotto buffer in TBS supplemented with 0.01% Tween-20 at 37°C for 1 hour. Consequently, plates were washed and incubated with a 4-fold dilution series of serum starting at a 1:40 dilution at 37°C for 2 hours. Following serum incubation, plates were washed and horseradish peroxidase (HRP)-labeled rabbit anti-human IgG (1:6,000, Dako) was added. Plates were incubated at 37°C for 1 hour, washed and developed with 3,3′,5,5′-tetramethylbenzidine (KPL). Signal was measured at an optical density of 450 nm (OD450) using an ELISA microtiter plate reader (infinite F200, Tecan). OD450 signal was corrected by subtracting background signal in the OD620 channel, a min-max S-curve was generated based on the lowest and highest OD450 value, and a 50% endpoint titer was calculated.

#### Antibody dependent cellular cytotoxicity (ADCC)

The presence of antibody dependent cell mediated cytotoxicity (ADCC)-mediating antibodies was determined in an established assay that measures NK92.05-CD16 cell degranulation.^62^ In short, high-binding 96-wells plates (Immunolon) were coated with baculovirus-generated trimeric prefusion His-tagged S protein (200 ng/well) from ancestral SARS-CoV-2 (D614G), and Delta and Omicron BA.1 variants (SinoBiologicals) at 4°C overnight. Plates were blocked, washed and incubated with serum (diluted 1:160 and 1:640) at 37°C for 2 hours. Following serum incubation, plates were washed and 100.000 NK92.05-CD16 cells were added, in combination with CD107a^V450^ (1:100, clone H4A3, BD), Golgistop (0.67 μL/mL, BD), and GolgiPlug (1 μL/mL, BD). Plates were incubated at 37°C for 5 hours, washed and stained for viable NK cells with CD56^PE^ (1:25, clone B159, BD) and LIVE/DEAD Fixable Aqua Dead Cell (AmCyan, Invitrogen, 1:100). Cells were stained at 4°C for 30 minutes and fixed in Cytofix/Cytoperm (BD Biosciences) at 4°C for 30 minutes. Activated NK92.05-CD16 cells were acquired in a FACSLyric (BD) and identified as CD56^+^CD107a^+^ cells. Gating strategy is depicted in Figure S12A. Percentages were corrected by subtracting background measured on PBS-coated plates. Two independent experiments were performed, one at a serum dilution of 1:160 (Figure S12B) and another at 1:640 (Figure S12C) because some samples showed a prozone effect at a 1:160 dilution (Figures S8D and S8E), which gave an underrepresentation of the ADCC signal. Average values were used for the main data (Figures 2B and 2C).

#### Antibody dependent cellular phagocytosis (ADCP)

The presence of antibody dependent cellular phagocytosis (ADCP)-mediating antibodies was determined in an assay that measures phagocytosis of S-coated fluorescent beads.^26^ The monocytic THP-1 cell line (ATCC, TIB-202™) was used to measure ADCP. Briefly, fluorescent Neutravidin beads (FluoSpheres, Life Technologies) were linked to biotin-labeled monomeric S protein from ancestral (D614G) SARS-CoV-2 (Sino Biologicals) by incubating 100 μL beads with 100 μg protein at 37°C for 2 hours. Sera was added to the S-coated FluoSphere beads in a 4-fold dilution series ranging from a 1:40 to 1:2,560 dilution, or 1:2,560 to 1:163,000 dilution in the case of a S1-specific antibody level >1000 BAU/mL, and incubated at 37°C for 2 hours. 50,000 THP-1 cells were added per well and incubated at 37°C overnight after which FluoSphere bead phagocytosis was measured as PE-positive THP-1 cells by flow cytometry in a FACSLyric (BD). Representative dilution series is shown in Figure S4A. ADCP percentages were corrected for PBS control and endpoint titers were determined at an arbitrary cut-off of 20%.

#### Detection of RBD-specific B cells by flow cytometry

RBD-specific B cells were measured using fluorescently labeled SARS-CoV-2 RBD-tetramers (SARS-CoV-2 RBD B cell analysis kit, Miltenyi Biotec). In brief, 8–10 × 10^6^ PBMC were incubated with recombinant SARS-CoV-2 RBD-tetramer^PE^ and RBD-tetramer^PE-Vio770^ to stain for RBD-specific B cells. Subsequently, the cells were stained with fluorescently labeled antibodies detecting CD19^APC−Vio770^ (clone LT19), CD27^Vio Bright FITC^ (clone M-T271), IgG^VioBlue^ (clone IS11-3B2.2.3), IgA^VioGreen^ (clone IS11-8E10) and IgM^APC^ (clone PJ2-22H3). Live-dead staining was performed using 7-AAD. Flowcytometry analysis of the whole sample was performed using the FACS Canto II (BD). The proportion of total RBD-specific B cells, RBD-specific memory B cells and RBD-specific IgG memory B cells were determined using FlowJo 10.8.1 (TreeStar, Ashland, OR, USA). The gating strategy is displayed in Figure S3A.

#### Detection of S-specific T cells by IFN-γ release assay

The presence of SARS-CoV-2-specific T cells was initially measured by a commercially available IFN-ɣ Release Assay (IGRA, QuantiFERON, Qiagen) in whole blood.^63^^,^^64^ Briefly, heparinized whole blood was incubated with three different SARS-CoV-2 antigens for 20–24 h using a combination of overlapping peptides stimulating both CD4 and CD8 T cells either representing a fraction of the S protein (Ag1), the entire S protein (Ag2), or a combination of specific peptides inherited from the full SARS-CoV-2 genome (Ag3). We focused on the Ag2 data in this manuscript as the peptide composition of this stimulation compares best to the overlapping peptide pools used in AIM and ELISpot. Mitogen-coated tubes were used as positive control and carrier coated tubes were included as negative control. After incubation, plasma was obtained by centrifugation and IFN-ɣ production in response to the antigens was measured by ELISA. Results were expressed in international units (IU) IFN-ɣ/mL after subtraction of the NIL control values as interpolated from a standard calibration curve. Lower limit of detection in this assay was set at 0.01 IU/mL, and the responder cut-off was set at 0.15 IU/mL per manufacturer’s instructions, as used in previous studies.^10^

#### Detection of S-specific T cells by IFNγ ELISPOT

SARS-CoV-2-specific T cells were measured using IFNγ ELISpot. In short, -multiscreen® HTS IP filter plates (Millipore) activated with 35% ethanol were coated with anti-human IFN-γ antibody (1-D1K, Mabtech; 5 μg/mL) and incubated overnight at 4°C. Next, plates were blocked with X-VIVO (Lonza) medium +2% Human AB Serum (HS; Sigma). PBMCs were thawed, resuspended in IMDM (Gibco) + 10% FCS, and washed twice. In X-VIVO +2%HS, PBMCs were brought to a concentration of 4 × 10^6^ cells/mL and rested for 1 hour at 37 °C. SARS-CoV-2 S1 and S2 peptide pools, (JPT Peptide Technologies) consisting of 15-mer peptides overlapping by 11 amino acids that cover the S protein were used for stimulation at a concentration of 0.5 ug/mL. All stimulation were performed in triplicate. 0.4% DMSO (Sigma) was used as negative control and PHA (Remel Europe Ltd; 4 μg/mL) as a positive control. 2 × 10^5^ PBMC were added per well and cultured for 20–24 hours at 37 °C. The next day, ELISpot plates were washed with PBS +0.05% Tween-20. Anti-human biotinylated IFN-γ antibody (7-B6-1, Mabtech; 1:1000) in 0.05% Poly-HRP buffer (ThermoFisher) was added for 1.5 hours at RT, followed by the addition of Streptavidin poly-HRP (Sanquin; 1:6000) in 0.05% Poly-HRP buffer for 1 hour at RT (in the dark). Spots were developed using TMB substrate (Mabtech). Spot forming cells (SFC) were quantified with the AID ELISpot/Fluorospot reader and calculated to SFCs/10^6^ PBMC. The average of the DMSO negative control was subtracted per stimulation. To define the total S-specific SFC, the sum of SFC of the separate S1 and S2 peptide pools was used. An antigen-specific response of ≥50 SFC/10^6^ PBMCs was considered positive. Samples were excluded when the positive PHA control was negative.

#### Detection of S-specific T cells by activation induced marker (AIM) assay

PBMC were thawed in RPMI1640 medium supplemented with 10% FBS, 100 IU/mL penicillin, and 100 IU/mL streptomycin (R10F) and incubated with Benzonase® (50 IU/mL; Merck) at 37°C for 30 minutes. Benzonase is an endonuclease that is added during the thawing process to improve PBMC viability. Subsequently, 1 × 10^6^ PBMC were incubated with in-house developed SARS-CoV-2 peptide pools (15mers with 10 overlaps, 1 μg/mL per peptide) covering the ancestral, Delta or Omicron BA.1 S protein at 37°C for 20 hours; PBMC were stimulated with an equimolar amount of DMSO as negative control or a combination of PMA (50 μg/mL) and Ionomycin (500 μg/mL) as positive control. Following stimulation, PBMC were stained for surface markers at 4°C for 15 minutes with the following antibodies in their respective dilutions: anti-CD3^PerCP^ (Clone SK7, BD, 1:25), anti-CD4^V450^ (Clone L200, BD, 1:50), anti-CD8^FITC^ (Clone DK25, Dako, 1:25), anti-CD45RA^PE−Cy7^ (Clone L48, BD, 1:50), anti-CCR7^BV711^, anti-CD69^APC−H7^ (Clone FN50, BD, 1:50), anti-CD137^PE^ (Clone 4B4-1, Miltenyi, 1:50), and anti-OX40^BV605^ (Clone L106, BD, 1:25). LIVE/DEAD™ Fixable Aqua Dead Cell staining was included (AmCyan, Invitrogen, 1:100). T cells were gated as LIVE CD3^+^ cells and subdivided into CD4^+^ or CD8^+^ subsets. Memory subsets were identified as either CD45RA^+^CCR7^+^ (naïve, T_N_), CD45RA^−^CCR7^+^ (central memory, T_CM_), CD45RA^−^CCR7^-^ (effector memory, T_EM_), or CD45RA^+^CCR7^-^ (terminally differentiated effectors, T_EMRA_). SARS-CoV-2-reactive T cells were identified as activated T cells after exclusion of T_N_ cells (CD137^+^Ox40^+^ for CD4^+^, or CD137^+^CD69^+^ for CD8^+^). The gating of subsets and activated cells was set based on the DMSO stimulated sample on a per donor basis. On average, 300,000 cells were acquired on a FACSLyric (BD). Samples with <50,000 counts in the CD3 gate were excluded from analysis. Gating strategy is depicted in Figure S6A. A LLoD of 0.01% was set to allow reproducible detection of AIM+ cells within the CD4^+^ or CD8^+^ gate.

#### T-cell receptor variable beta chain sequencing

Genomic DNA (gDNA) was extracted from 1 × 10^6^ PBMC using the DNeasy Blood Extraction Kit (QIAGEN). Depending on the yield, between 12 and 375 μg gDNA was used for immunosequencing of the CDR3 regions of the TCRβ chain by the immunoSEQ® Assay (Adaptive Biotechnologies, Seattle, WA). Extracted gDNA was amplified in a bias-controlled multiplex PCR, followed by high-throughput sequencing. Obtained sequences were collapsed and filtered to identify the absolute abundance of each unique TCR TCRβ CDR3 region for further analysis. TCR sequences from repertoires were mapped against a set of TCR sequences that are known to react with SARS-CoV-2 by matching on V gene, amino acid sequence and J gene. In brief, these sequences were first identified by Multiplex Identification of T-cell Receptor Antigen Specificity (MIRA, Klinger et al., 2015). The COVID-19 search tool from immunoSEQ was used to identify these SARS-CoV-2-specific T cell clones, and the number of agnostic expanded T-cell clones following booster vaccination was estimated using the differential abundance tool from ImmunoSEQ.^16^ Individual responses were quantified by the number and frequency of SARS-CoV-2 TCRs. These were further analyzed at the level of ORF or position within ORF based on the MIRA antigens. The breadth was calculated as the number of unique annotated rearrangements out of the total number of productive rearrangements, while the depth was calculated as the sum frequency of those rearrangements in the repertoire. Two samples were excluded from further analysis due to quality of the sample or gDNA cross-contamination.

### Quantification and statistical analysis

The baseline characteristics in each group (Ad26.COV2.S/no boost, Ad26.COV2.S/Ad26.COV2.S, Ad26.COV2.S/mRNA-1273, and Ad26.COV2.S/BNT162b2) are described in Table 1. Categorical variables are presented as numbers and percentages (%). Differences between the groups were compared with the use of Fisher’s exact test.

Continuous variables were presented as medians and interquartile ranges or individual datapoints. Kruskal-Wallis test followed by Dunn’s multiple comparisons was performed for comparison of binding antibodies (ELISA and Liaison), ADCC-mediating antibodies, ADCP-mediating antibodies, neutralizing antibodies, IGRA, IFNg ELISpot, and B-cell flow cytometry between groups; only differences between Ad26.COV2.S and mRNA-1273, or Ad26.COV2.S and BNT162b2 are shown in the figures. Friedman test followed by Dunn’s multiple comparisons was used to compare binding antibodies, ADCC-mediating antibodies, and neutralizing antibodies to variants within each group; only differences between ancestral SARS-CoV-2 and variants are shown in the figures. Wilcoxon rank test was performed for the comparison of pre- versus post-booster vaccination responses.

Limited material was available for the AIM assay, leading to incomplete datasets for these read-outs. Therefore, for statistical analysis of SARS-CoV-2-specific T-cell responses determined by AIM, Mann-Whitney U test was performed for comparison of vaccine groups Ad26.COV2.S and mRNA-1273, and Ad26.COV2.S and BNT162b2; a p-value of 0.025 was considered significant after Bonferroni correction. Wilcoxon rank test was performed for the comparison of variant-specific T-cell responses between ancestral SARS-CoV-2 and variants; a p-value of 0.025 was considered significant after Bonferroni correction. Wilcoxon rank test was performed for the comparison of pre- versus post-booster vaccination responses for both CD4 and CD8 T-cells; a p-value of 0.05 was considered significant.

To examine associations between two continuous variables, we estimated a Spearman’s correlation coefficient.

## Data Availability

• All data reported in this paper will be shared by the lead contact upon request. This work is licensed under a Creative Commons Attribution 4.0 International (CC BY 4.0) license, which permits unrestricted use, distribution, and reproduction in any medium, provided the original work is properly cited. To view a copy of this license, visit https://creativecommons.org/licenses/by/4.0/. This license does not apply to figures/photos/artwork or other content included in the article that is credited to a third party; obtain authorization from the rights holder before using such material. • This paper does not report original code. • Any additional information required to reanalyze the data reported in this paper is available from the lead contact upon request.
