# Supplementary material for: Ad26.COV2.S priming provided a solid immunological base for mRNA-based COVID-19 booster vaccination
Source: iScience. 2022 Dec 7;26(1):105753. doi: 10.1016/j.isci.2022.105753 (PMC9726653; doi:10.1016/j.isci.2022.105753)
Supplement: Document S1. Figures S1–S12 [file mmc1.pdf]

iScience, Volume *26*

Supplemental information

Ad26.COV2.S priming provided a solid immunological base for mRNA-based COVID-19 booster vaccination

Daryl Geers, Roos S.G. Sablerolles, Debbie van Baarle, Neeltje A. Kootstra, Wim J.R. Rietdijk, Katharina S. Schmitz, Lennert Gommers, Susanne Bogers, Nella J. Nieuwkoop, Laura L.A. van Dijk, Eva van Haren, Melvin Lafeber, Virgil A.S.H. Dalm, Abraham Goorhuis, Douwe F. Postma, Leo G. Visser, Anke L.W. Huckriede, Alessandro Sette, Alba Grifoni, Rik L. de Swart, Marion P.G. Koopmans, P. Hugo M. van der Kuy, Corine H. GeurtsvanKessel, Rory D. de Vries, and on behalf of the SWITCH research group

Supplemental Appendix

Table of contents

List of investigators	2
Supplemental Figure 1	3
Supplemental Figure 2	4
Supplemental Figure 3	5
Supplemental Figure 4	6
Supplemental Figure 5	7
Supplemental Figure 6	8
Supplemental Figure 7	9
Supplemental Figure 8	10
Supplemental Figure 9	11
Supplemental Figure 10	12
Supplemental Figure 11	13
Supplemental Figure 12	14

SWITCH research group

Erasmus MC

N. Tjon

K. van Grafhorst

L.P.M. van Leeuwen

F. de Wilt

S. Scherbeijn

A.C.P. Lamoré

Amsterdam UMC

H.M. Garcia Garrido

A.M. Harskamp

I. Maurer

A.F. Girigorie

B. D. Boeser-Nunnink

M.M. Mangas Ruiz

K. A. van Dort

UMCG

J.J. de Vries-Idema

J. Zuidema

LUMC

J.A. Vlot

P.H. Verbeek –Menken

A.Van Wengen-Stevenhagen

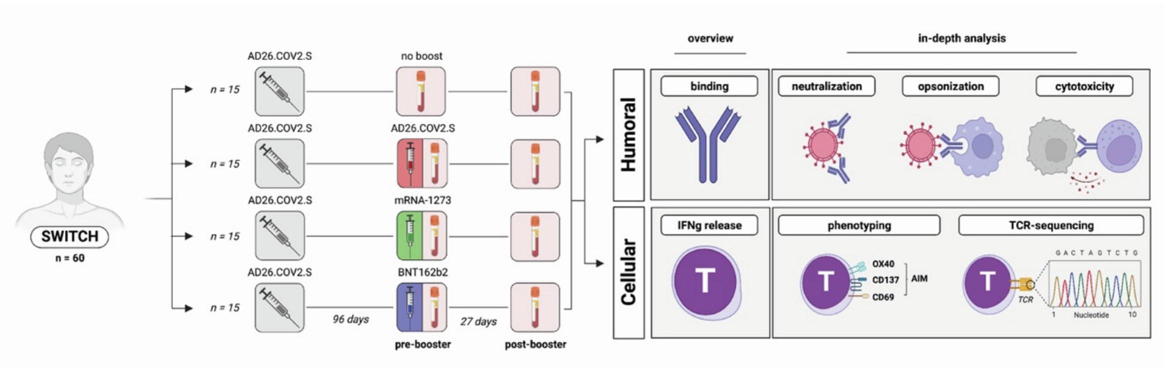

Figure S1: Study design and previously reported binding antibody levels and whole blood T-cell responses. Study design of the SWITCH immune profiling analysis. Immune responses in n=60 individuals that received either no (grey), Ad26.COV2.S (red), mRNA-1273 (green), or BNT162b2 (blue) booster vaccination were assessed pre- and post-booster vaccination.

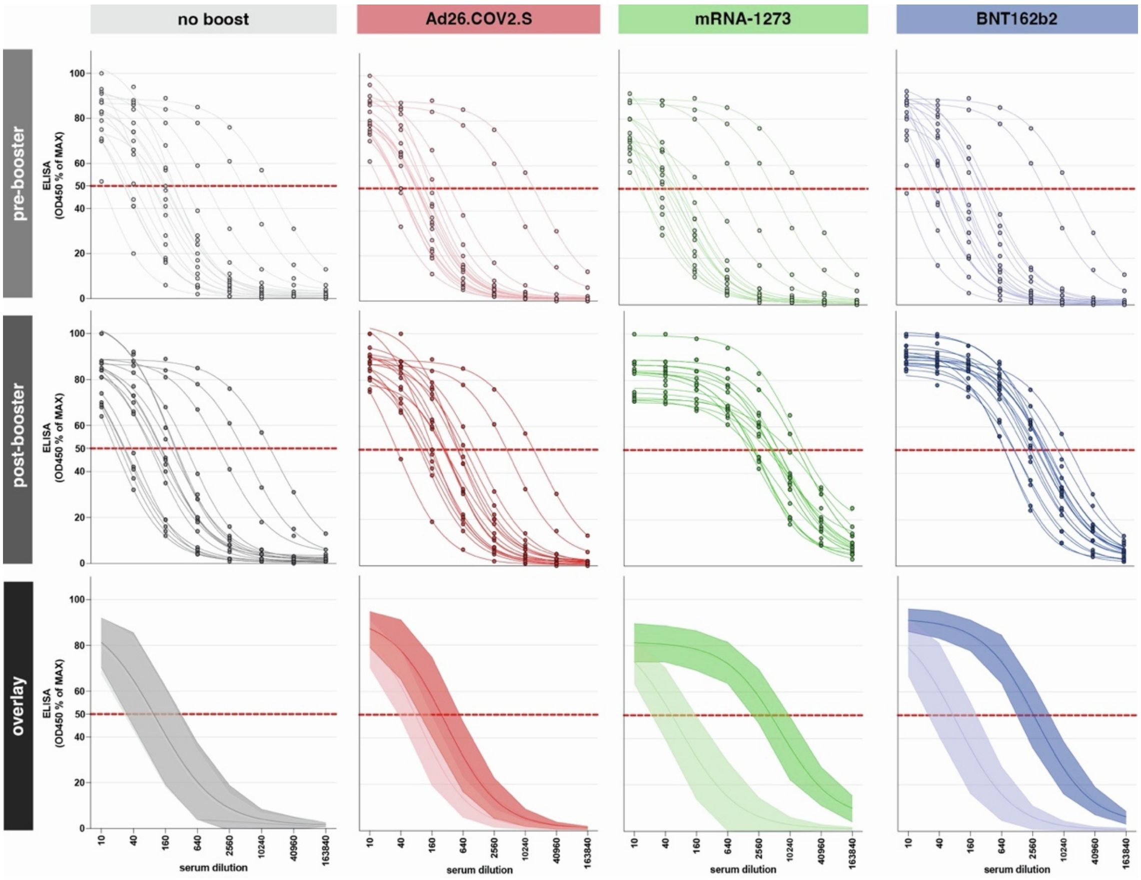

Figure S2: ELISA S-curves per vaccination regimen. Related to Figure 1. Log(inhibitor) versus response curves with four parameter variable slopes based on OD450 values % of max per vaccine group, pre-booster vaccination (top), post-booster vaccination (middle), and overlayed depicting the group mean with error bands representing the standard deviation (bottom). Red dotted line indicates the 50% endpoint.

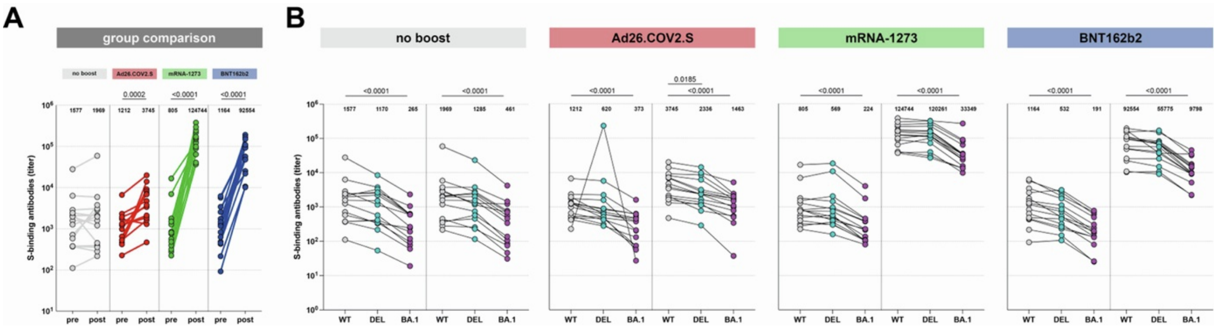

Figure S3: Binding antibody levels pre- and post-booster vaccination. Related to Figure 1. (A) S-specific binding antibody levels following no boost (grey), Ad26.COV2.S (red), mRNA-1273 (green), or BNT162b2 (blue) vaccination following Ad26.COV2.S priming. **(B)** Cross-reactivity of S-specific binding antibodies against ancestral SARS-CoV-2 (grey), Delta (cyan), or Omicron BA.1 (pink) variants pre- and post-booster. WT = ancestral virus, delta = Delta variant, BA.1 = Omicron BA.1 variant. Symbols represent individual donors (n=15 per group). Wilcoxon rank test was performed for the comparison of pre- versus post-booster vaccination responses; a p-value of 0.05 was considered significant. Friedman test followed by Dunn's multiple comparisons was used to compare vaccine responses to variants within each group; only differences between ancestral SARS-CoV-2 and variants are shown in the figure. This supplemental figure is an alternative illustration of the data depicted in **Fig. 1B** and **1C**.

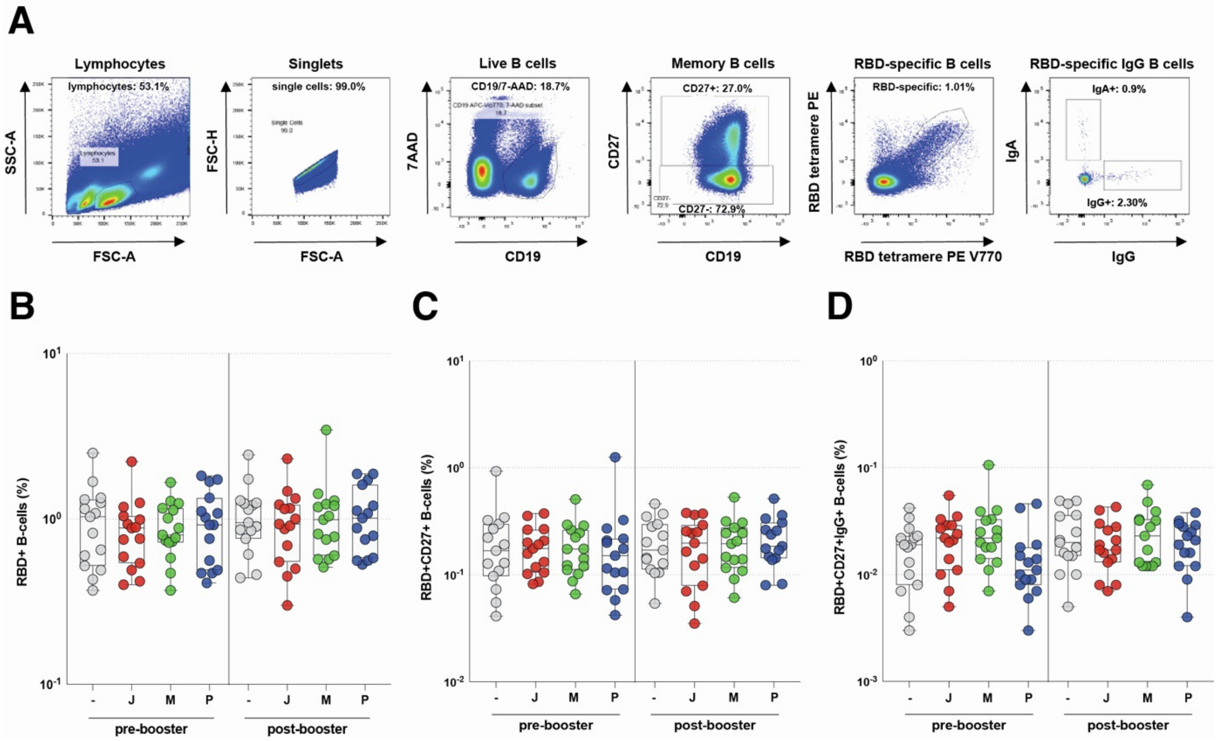

Figure S4: Gating strategy and detection of RBD-specific B-cells. Related to Figure 1. (A) Lymphocytes were gated based on the SSC-A and FSC-A and single cells were selected based on FSC-H and FSC-A. Next, LIVE CD19+ cells were selected for the analysis of RBD-specific B cells. In addition, the CD27+ subpopulation of the CD19+ B-cells (memory B cells) and the CD27+ IgG+ subpopulation was selected. In each subpopulation, the proportion of RBD-specific B cells was determined by gating on RBD-tetramer-PE and RBD-tetramer-PE-Vio770 double positive cells. **(B)** Percentage of total RBD-specific B cells, **(C)** RBD-specific memory B cells, and **(D)** RBD-specific IgG memory B cells of total B cells in whole blood after no-boost (grey), Ad26.COV2.S (red), mRNA-1273 (green), or BNT162b2 (blue) booster vaccination pre- and post-booster vaccination. - = no boost, J = Ad26.COV2.S, M = mRNA-1273, P = BNT162b2. Symbols represent individual donors (n=15 per group). Kruskal-Wallis test followed by Dunn’s multiple comparisons was performed for comparison of vaccine responses between groups; only differences between Ad26.COV2.S and mRNA-1273, or Ad26.COV2.S and BNT162b2 are shown in the figure.

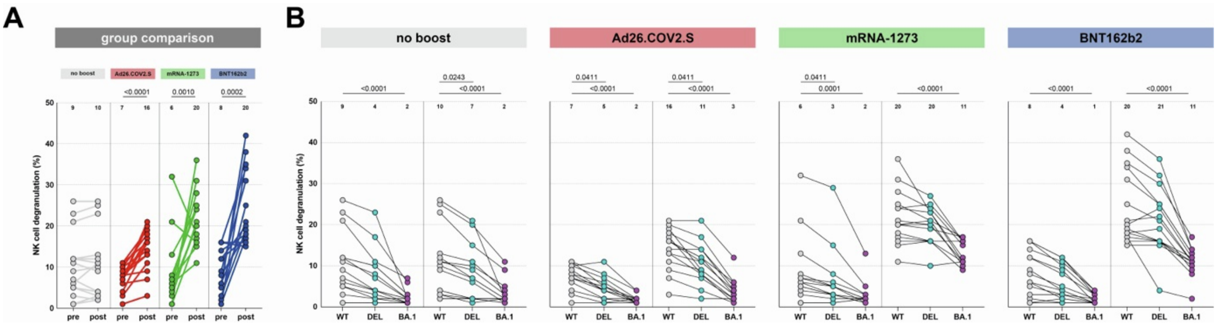

Figure S5: ADCC-mediating antibody levels pre- and post-booster vaccination. Related to Figure 2. (A) S-specific ADCC-mediating antibody levels following no boost (grey), Ad26.COV2.S (red), mRNA-1273 (green), or BNT162b2 (blue) vaccination following Ad26.COV2.S priming. **(B)** Cross-reactivity of S-specific ADCC-mediating antibodies against ancestral SARS-CoV-2 (grey), Delta (cyan), or Omicron BA.1 (pink) variants pre- and post-booster. WT = ancestral virus, delta = Delta variant, BA.1 = Omicron BA.1 variant. Symbols represent individual donors (n=15 per group). Wilcoxon rank test was performed for the comparison of pre- versus post-booster vaccination responses; a p-value of 0.05 was considered significant. Friedman test followed by Dunn's multiple comparisons was used to compare vaccine responses to variants within each group; only differences between ancestral SARS-CoV-2 and variants are shown in the figure. This supplemental figure is an alternative illustration of the data depicted in **Fig. 2B** and **2C**.

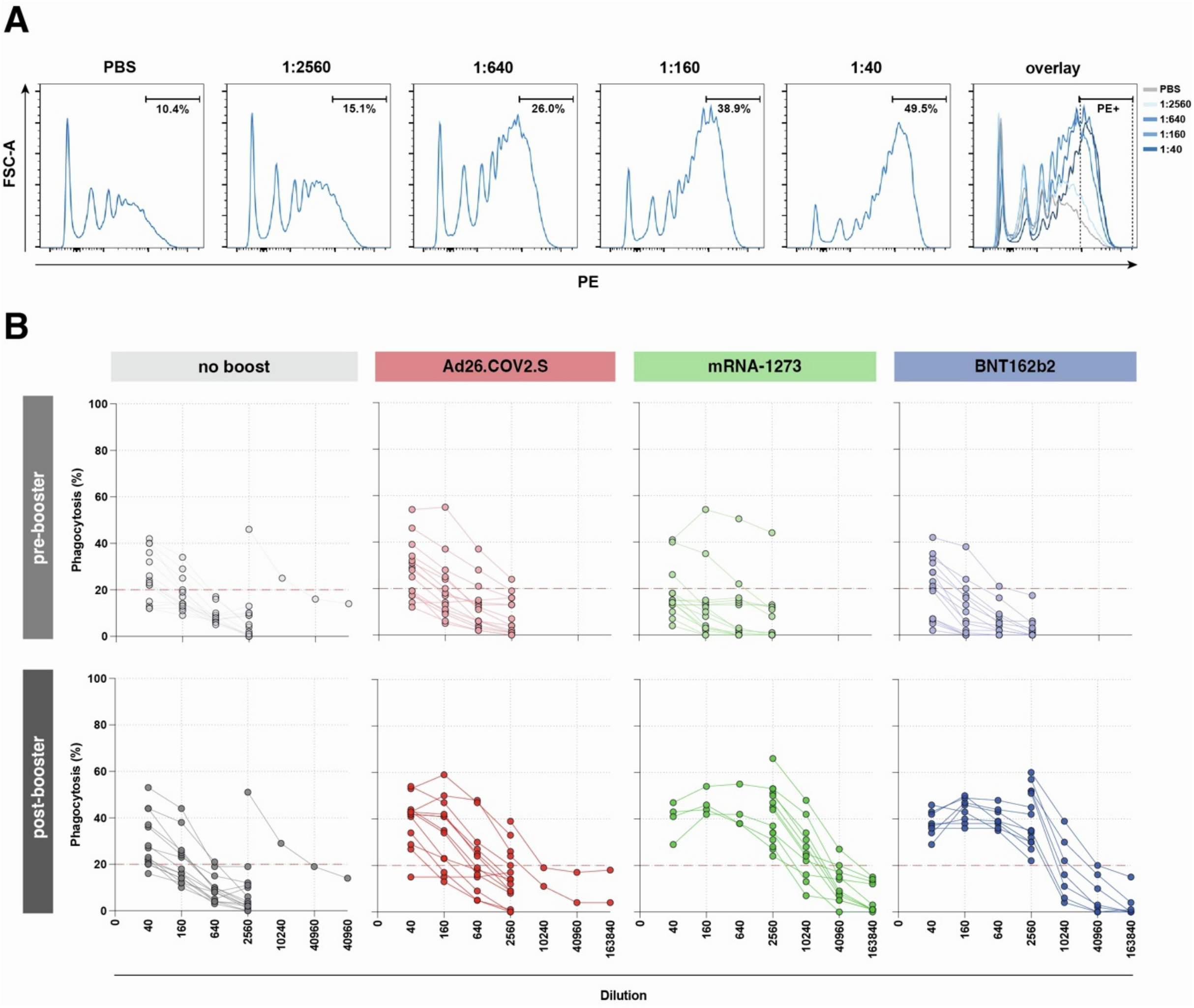

Figure S6: ADCP selection of PE+ cells in a dilution series from one representative sample and individual ADCP S-curves per vaccine regimen. Related to Figure 2. (A) Selection of phagocytosing cells. PE signal in PBS control was set at 10% background phagocytosis, and positive sera diluted 1:2560, 1:640, 1:160, and 1:40 from left to right. Overlay depicts PBS control in grey and different dilutions of sera in shades of blue. **(B)** Individual dilution series in ADCP pre- and post-booster vaccination per group: 'no boost' (grey), Ad26.COV2.S boost (red), mRNA-1273 boost (green), and BNT162b2 boost (blue). Red dotted line indicates the 20% endpoint dilution, which was used for calculation of ADCP-mediating antibody titers.

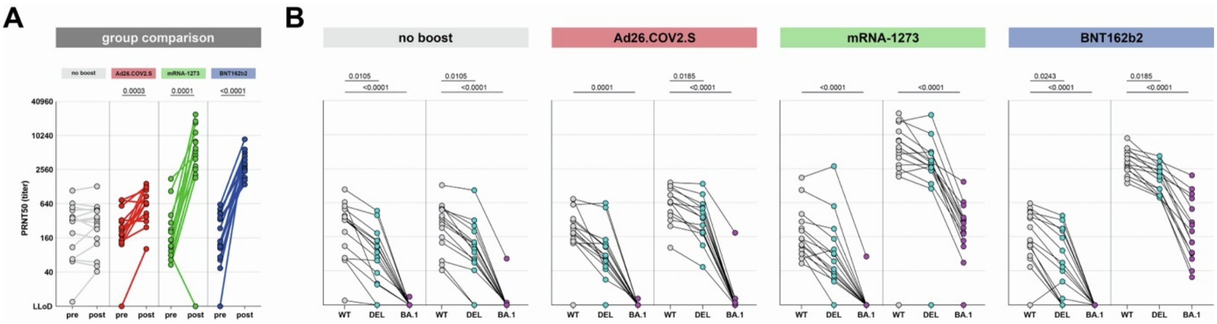

Figure S7: Neutralizing antibody levels pre- and post-booster vaccination. Related to Figure 3. (A) Neutralizing antibody levels following no boost (grey), Ad26.COV2.S (red), mRNA-1273 (green), or BNT162b2 (blue) vaccination following Ad26.COV2.S priming. **(B)** Cross-reactivity of neutralizing antibodies against ancestral SARS-CoV-2 (grey), Delta (cyan), or Omicron BA.1 (pink) variants pre- and post-booster. WT = ancestral virus, delta = Delta variant, BA.1 = Omicron BA.1 variant. Symbols represent individual donors (n=15 per group). Wilcoxon rank test was performed for the comparison of pre- versus post-booster vaccination responses; a p-value of 0.05 was considered significant. Friedman test followed by Dunn's multiple comparisons was used to compare vaccine responses to variants within each group; only differences between ancestral SARS-CoV-2 and variants are shown in the figure. This supplemental figure is an alternative illustration of the data depicted in **Fig. 3B** and **3C**.

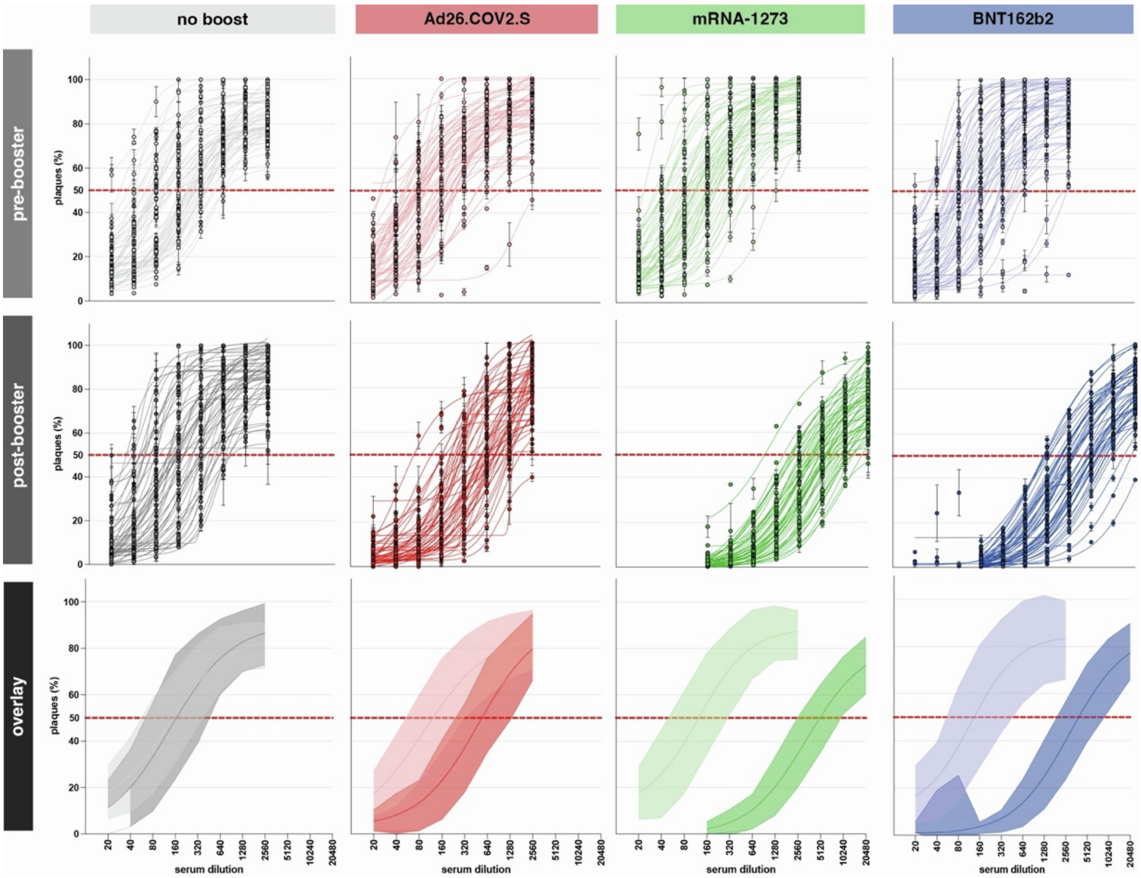

Figure S8: Individual PRNT S-curves per vaccine regimen. Related to Figure 3. log(inhibitor) versus response curves with four parameter variable slopes based on plaque counts compared to the virus control per vaccine group, pre-booster vaccination (top), post-booster vaccination (middle), and overlayed depicting the group mean with error bands representing the standard deviation (bottom). Red dotted line indicates the 50% endpoint.

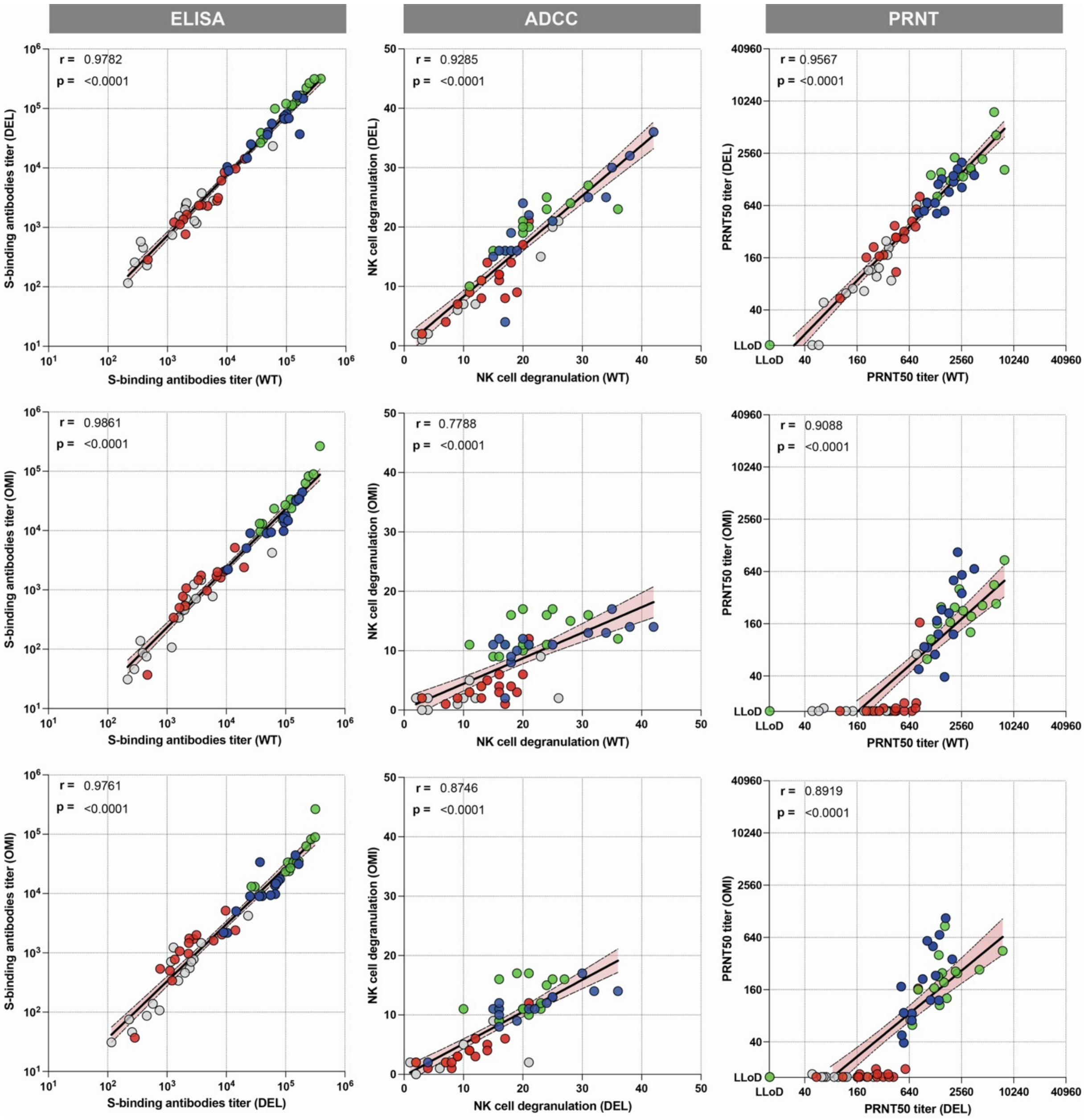

Figure S9: Binding and functional antibody responses to variants are correlated. Related to Figure 4. Correlation between S-specific binding, ADCC-mediating and neutralizing antibodies between ancestral (WT) and delta (DEL), ancestral and omicron BA.1 (OMI), or delta and omicron BA.1. Simple linear regression analysis on log-transformed data was used to calculate Spearman's correlation coefficient and p-values. Colors represent different booster groups: no boost (grey), Ad26.COV2.S boost (red), mRNA-1273 boost (green), and BNT162b2 boost (blue).

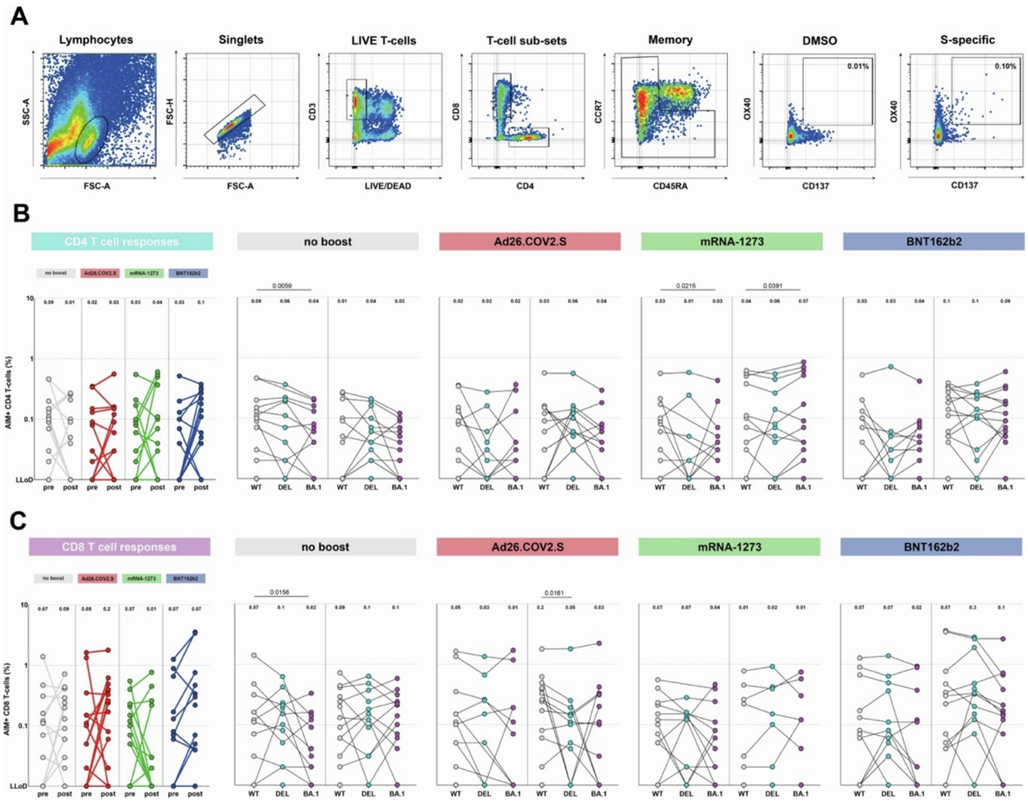

Figure S10: T cell responses pre- and post-booster vaccination. Related to Figure 5. (A) Lymphocytes were gated based on the SSC-A and FSC-A and single cells were selected based on FSC-H and FSC-A. Next, LIVE CD3+ cells were selected and sub-divided into CD4+ or CD8+ T-cells. From each sub-population naïve T-cells are excluded from further analysis based on their expression of CCR7 and CD45RA. Within the memory T-cell population OX40+CD137+ or CD69+CD137+ CD4 or CD8 T-cells, respectively, are defined as activated. A representative DMSO stimulated and S stimulated sample is shown. **(B)** CD4 T-cell responses following no boost (grey), Ad26.COV2.S (red), mRNA-1273 (green), or BNT162b2 (blue) vaccination following Ad26.COV2.S priming. Cross-reactivity of CD4 T-cells against ancestral SARS-CoV-2 (grey), Delta (cyan), or Omicron BA.1 (pink) variants pre- and post-booster (right panels). **(C)** CD8 T-cell responses following no boost (grey), Ad26.COV2.S (red), mRNA-1273 (green), or BNT162b2 (blue) vaccination following Ad26.COV2.S priming. Cross-reactivity of CD8 T-cells against ancestral SARS-CoV-2 (grey), Delta (cyan), or Omicron BA.1 (pink) variants pre- and post-booster (right panels). WT = ancestral virus, delta = Delta variant, BA.1 = Omicron BA.1 variant. Symbols represent individual donors. Wilcoxon rank test was performed for the comparison of pre- versus post-booster vaccination responses for both CD4 and CD8 T-cells; a p-value of 0.05 was considered significant. Wilcoxon rank test was performed for the comparison of variant-specific T-cell responses between ancestral SARS-CoV-2 and variants; a p-value of 0.025 was considered significant after Bonferroni correction. Supplemental figure 10B is an alternative illustration of the data depicted in **Fig. 5C** and **5D**.

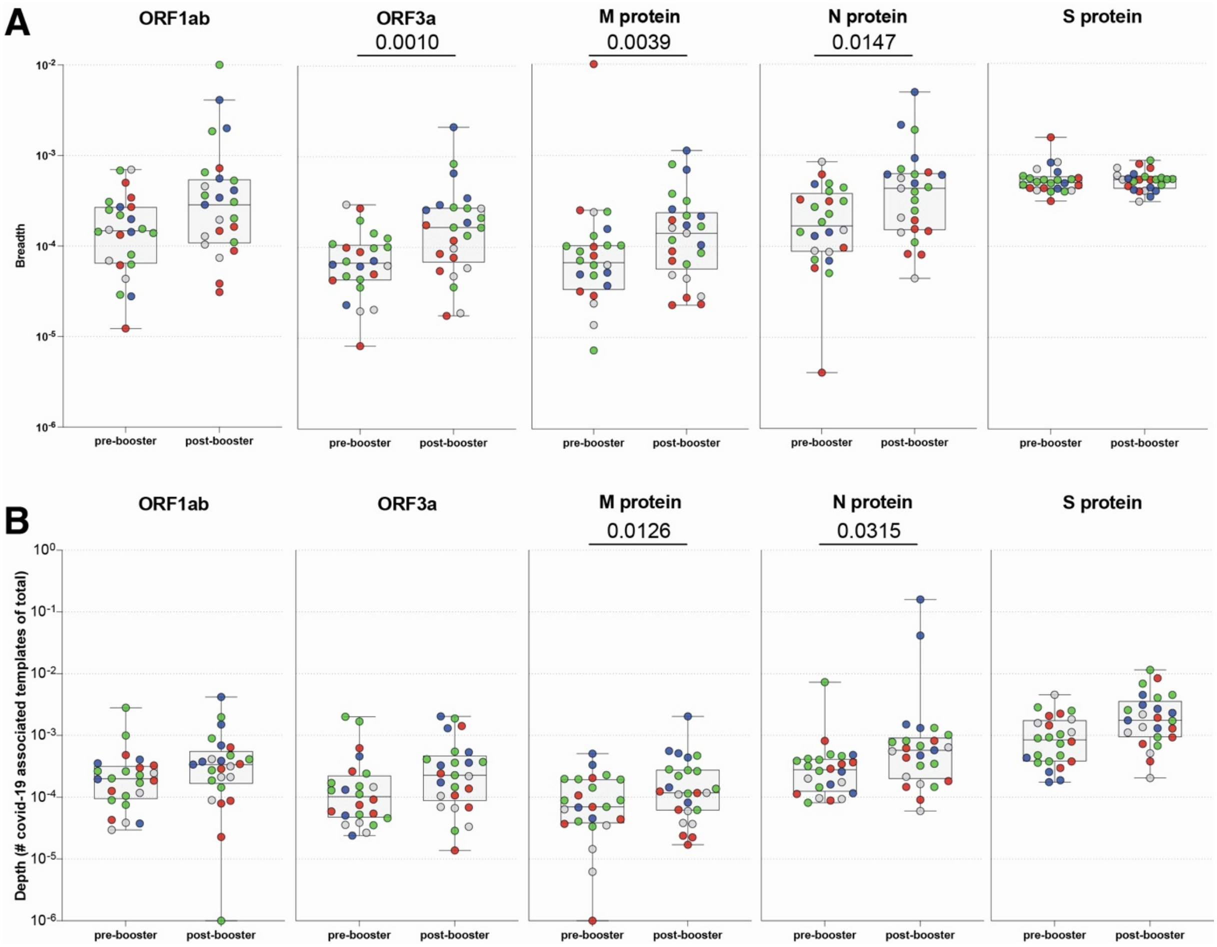

Figure S11: Breadth and depth of SARS-CoV-2-specific T-cell response. Related to Figure 6. (A) Breadth and **(B)** depth of the T-cell response to ORF1ab, ORF3a, M protein, N protein, and S protein pre- and post-booster vaccination. - = no boost (n=4 pre-boost / n=5 post-boost), J = Ad26.COV2.S (n=6), M = mRNA-1273 (n=10 pre-boost / n=8 post-boost), P = BNT162b2 (n=4 pre-boost / n=6 post-boost). Symbols represent individual donors. Box plot depicts the median with range (min to max). Wilcoxon rank test was performed for the comparison of T-cell breadth and depth pre- and post-booster vaccination.

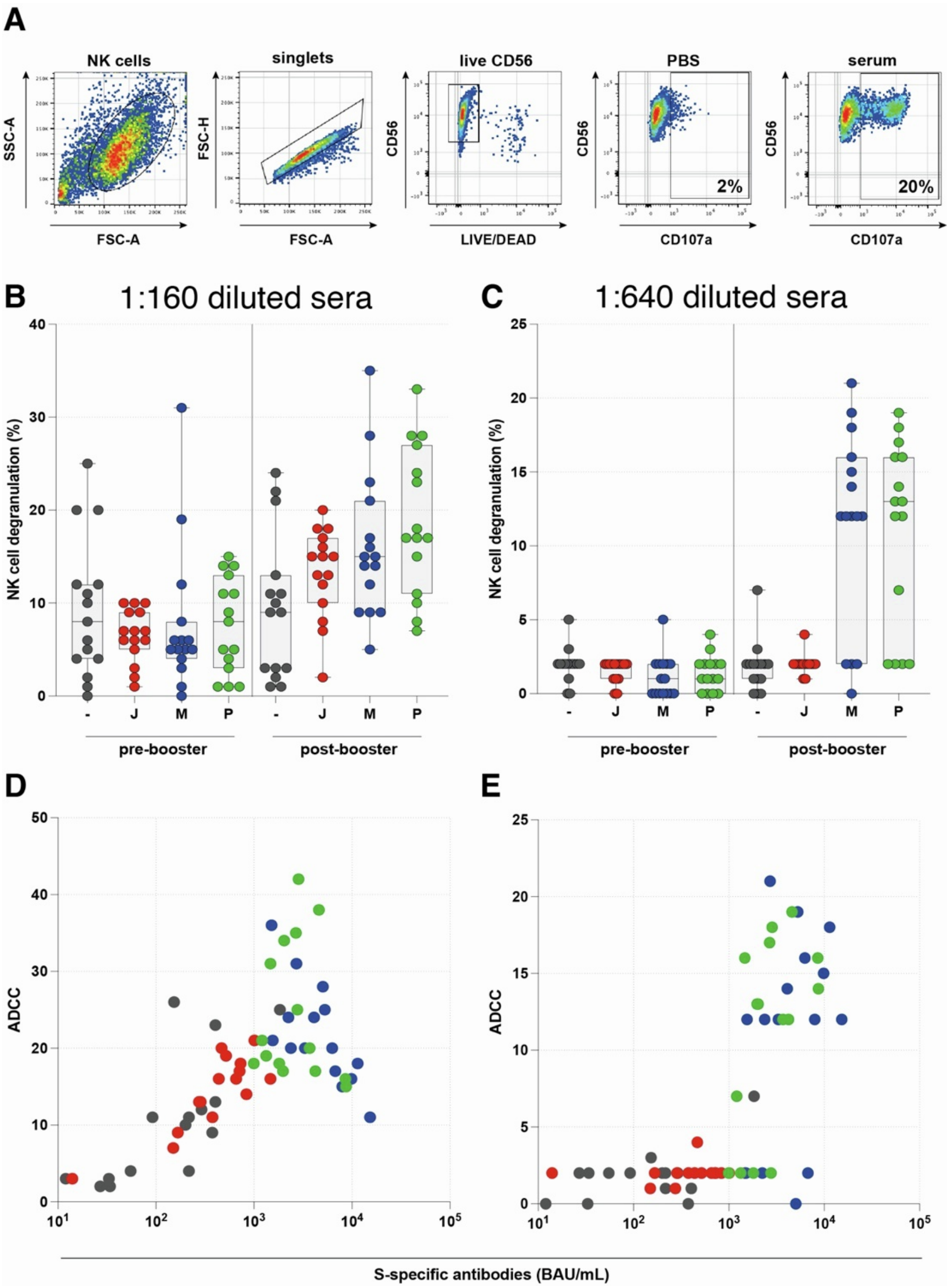

Figure S12: ADCC gating strategy and measurements from two independent experiments at two different serum dilutions. Related to Figure 2. (A) Gating strategy for ADCC assay, NK cells were gated on FSC-A and SSC-A, next singlets were selected by FSC-H and FSC-A and LIVE $CD56^{+}$ cells were gated to assess CD107a expression. Representative figure for a PBS and positive serum sample after booster vaccination with mRNA-1273 are shown. **(B)** ADCC data with serum samples diluted 1:160 and **(C)** 1:640. NK cell degranulation is depicted as a % after PBS subtraction pre- and post-booster vaccination after 'no boost' (grey), Ad26.COV2.S boost (red), mRNA-1273 boost (green), or BNT162b2 boost (blue). **(D)** correlation of ADCC inducing antibodies and S1-specific binding antibodies at 1:160 dilution and **(E)** 1:640 dilution. Symbols represent individual donors (n=15). Box plot depicts the median with range (min to max).
